# Single-cell sequencing of facial adipose tissue unveils FKBP5 as a therapeutic target for facial infiltrating lipomatosis

**DOI:** 10.1186/s13287-024-03835-9

**Published:** 2024-07-18

**Authors:** Hongrui Chen, Bin Sun, Shih-Jen Chang, Zhang Yu, Yajing Qiu, Chen Hua, Xiaoxi Lin

**Affiliations:** Department of Plastic & Reconstructive Surgery, Shanghai Ninth People’s Hospital, Shanghai Jiao Tong University School of Medicine, 639 Zhizaoju Road, Shanghai, 200011 P.R. China

**Keywords:** Facial infiltrating lipomatosis, Phosphatidylinositol 3-kinase catalytic subunit alpha, Fibro-adipogenic precursor cells, FK506 binding protein 51

## Abstract

**Background:**

Facial infiltrating lipomatosis is characterized by excessive growth of adipose tissue. Its etiology is associated with somatic phosphatidylinositol 3-kinase catalytic subunit alpha (PIK3CA) variants, but the specific mechanisms are not yet fully understood.

**Methods:**

We collected facial adipose tissue from both FIL patients and non-FIL individuals, isolated the stromal vascular fraction (SVF) and performed single-cell transcriptome sequencing on these samples.

**Results:**

We mapped out the cellular landscape within the SVF, with a specific focus on a deeper analysis of fibro-adipogenic precursor cells (FAPs). Our analysis revealed that FAPs from FIL patients (FIL-FAPs) significantly overexpressed FK506 binding protein 51 (FKBP5) compared to FAPs from individuals without FIL. Further experiments indicated that FKBP5 is regulated by the PI3K-AKT signaling pathway. The overactivation of this pathway led to an increase in FKBP5 expression. In vitro experiments demonstrated that FKBP5 promoted adipogenic differentiation of FAPs, a process that could be hindered by FKBP5 knockdown or inhibition. Additionally, in vivo assessments confirmed FKBP5’s role in adipogenesis.

**Conclusions:**

These insights into the pathogenesis of FIL underscore FKBP5 as a promising target for developing non-surgical interventions to manage the excessive adipose tissue growth in FIL.

**Supplementary Information:**

The online version contains supplementary material available at 10.1186/s13287-024-03835-9.

## Introduction

Facial infiltrating lipomatosis (FIL) is a congenital maxillofacial deformity characterized by unilateral subcutaneous adipose tissue overgrowth. Histologically, it is marked by mature, non-encapsulated adipocytes that can diffusely infiltrate the adjacent soft tissues [1]. The condition is visible at birth, with patients exhibiting hemifacial swelling and myriad phenotypic features such as macrodontia, hemimacroglossia, lip hypertrophy, epidermal nevi, and mucosal neuromas [2]. These abnormalities can significantly impact facial appearance. The infiltration of adipose tissue into surrounding muscles and glands can cause functional disruptions, impairing physiological activities such as mastication, swallowing, and speech [3]. Currently, there are no effective therapeutic strategies for FIL. Although surgical intervention can aid in the removal of lipomatosis and aesthetic reconstruction, radical excision is challenging due to the infiltrative nature of the adipose tissue. It is reported that the postoperative recurrence rate of FIL exceeds 60% [4]. Moreover, surgical complications such as facial nerve damage and postoperative scar formation cannot be overlooked [5]. Therefore, exploring the pathogenesis and developing non-surgical treatment strategies are of great significance for the clinical management of FIL.

The PI3K-AKT-mTOR pathway plays a crucial role in cellular activities, involved in regulating proliferation, differentiation, apoptosis, and more [6]. Recently, somatic (phosphatidylinositol 3-kinase catalytic subunit alpha) PIK3CA variants have been detected in the adipose tissue of FIL patients [7, 8]. PIK3CA encodes the p110α catalytic subunit of PI3Kα. PIK3CA variants can result in a structural alteration in p110α, leading to its release from the inhibitory effects of the p85α regulatory subunit, thereby continuously stimulating downstream signaling molecules and causing overactivation of the PI3K-AKT-mTOR pathway [9]. Our previous work found that PIK3CA hotspot variants (E542K, E545K, H1047R) can lead to more severe FIL phenotypes, characterized by an increased number and severity of affected soft tissues [2]. However, although PIK3CA variants may be the primary cause of FIL, it is still unclear how they initiate these pathological processes by impacting downstream molecules.

Adipose tissue is a highly plastic organ that participates in regulating endocrine activities, energy storage, and metabolic homeostasis. The expansion of adipose tissue primarily occurs via increased adipocyte size (hypertrophy) and/or number (hyperplasia) [10]. Besides adipocytes, various other cells reside in the surrounding connective tissue, including immune cells, vascular cells (e.g., endothelial cells, mural cells), and fibro-adipogenic precursor cells (FAPs) capable of differentiating into mature adipocytes. All non-adipocyte cellular components are collectively referred to as the stromal vascular fraction (SVF), which can be isolated through enzymatic digestion [11]. In this context, FAPs act as a supplementary source of adipocytes. Their abnormal proliferation and differentiation are closely linked to the lipoma formation [12]. However, our understanding of how FAPs change in FIL and contribute to disease progression is still limited.

The advent of single-cell RNA sequencing/single-cell transcriptome (scRNA-seq) provides unprecedented opportunities for a comprehensive and accurate understanding of cellular and molecular changes in both disease and healthy conditions. Unbiased high-throughput sequencing of SVF at the single-cell level has illuminated the cellular changes occurring within adipose tissue during disease progression. For instance, scRNA-seq revealed a significant increase of CLEC3B + FAPs in adipose tissue of individuals with lymphedema, identifying CLEC3B as a therapeutic target to counteract fibrosis [13]. In cachexia, there has been a noted reduction in the expression of genes that primarily regulate adipocyte differentiation within FAP populations, alongside an elevated expression of inflammatory response genes [14]. However, no studies have dissected the FAPs alteration in the adipose tissue of FIL.

In this study, we performed the first comprehensive and unbiased scRNA-seq analysis of SVF in both FIL and normal facial adipose tissue. We found that FK506 binding protein 51 (FKBP5) is relatively highly expressed in FAPs derived from FIL (FIL-FAPs). We validated that the expression of FKBP5 was regulated by the PI3K-AKT pathway, underscoring its crucial role in the adipogenic differentiation of FAPs. Reducing or inhibiting FKBP5 can suppress adipogenesis of FIL-FAPs. Overall, our study offers novel insights into the pathogenesis of FIL and reveals potential therapeutic strategies.

## Methods

The work has been reported in line with the ARRIVE guidelines 2.0.

### Human subject

Adipose tissues from four FIL patients and three patients underwent facial cutaneous lesions were included in the single-cell sequencing experiments. The patients’ clinical characteristics were presented in Additional file 1. Table S1. Adipose tissues from another 13 FIL patients were used for immunohistochemistry analyses. This study was performed in accordance with the Declaration of Helsinki and approved by the Ethics Committee of Shanghai Ninth People’s Hospital. The approval number was No.SH9H-2022-T215-1. FAPs were isolated and collected from adipose tissue, with written consent of donors.

### Mice

Procedures involving animals were approved by the Animal Experimentation Ethics Committee at Shanghai Ninth People’s Hospital. A suspension of 1 × 10^6 FIL-FAPs cells in 50 μL DPBS was thoroughly combined with 50 μL of Matrigel (Corning) for each implant. Both cell types were pre-treated with adipogenic induction medium for one day prior to mixing. The mice were anesthetized by isoflurane at a flow rate of 1 L/min using rodent anesthesia machine (Yuyan Corporation, China). This combined cell/Matrigel solution (100 μL for each implant) was subcutaneously administered into the dorsal region of 6-week-old female BALB/C nude mice, sourced from Shanghai SLAC Laboratory Animal Co. These mice were then individually housed under a 12-hour light/dark cycle, with access to standard diet and water. After 28 days, the mice were euthanized through cervical dislocation, and the implants were collected, being carefully dissected free from adjacent tissues. The FAPs subjected to each different treatment were injected subcutaneously into four separate mice, with a total of 28 nude mice being used in the experiment. The formula for calculating tumor volume (in mm^3) was: (shortest diameter)^2 × (longest diameter) × 0.5.

### FAPs isolation and culture

Adipose tissue was collected during operation. Prior to procurement, no medical treatments were administered to the samples. Immediately after collection, samples were cooled on ice for transport to the research facility. Subsequently, the adipose tissue was rinsed in phosphate-buffered saline (PBS) and sectioned into pieces measuring between 0.1 and 0.2 cm. These segments were combined with an equal volume of a serum-free medium (Cyagen, USA, HUXMD-90,062) that included 0.2% collagenase I (Worthington, USA, LS004196). This mixture underwent shaking incubation at 37 °C for 1 h at 150 rpm, followed by centrifugation at 1000 rpm for 5 min, after which the supernatant and fat layer were discarded. To lyse red blood cells, 30 mL of lysis buffer (Solarbio, CHN, R1010) was added, and the mixture was centrifuged again at 1000 rpm for 5 min. After discarding the supernatant, the pellet was resuspended in 10 mL of basal growth medium (Cyagen, USA, HUXMD-90,011) and thoroughly mixed. The suspension was then strained through a 100 μm cell strainer into a 10 cm culture dish and maintained in an incubator set to 37 °C with 5% CO2. Cells between the third and fifth passages were selected for use in subsequent experiments.

### Characterization of FAPs

The basal medium was replaced every 2 days. Upon reaching 80% confluence, both primary FIL-FAPs and CON-FAPs were washed twice with PBS and digested with two milliliters of trypsin (Gibco, USA, 25,200,072) for 2 min. Then, two milliliters of basal medium were added to stop digestion. The cell suspension was centrifuged for 5 min at 1000 rpm and the supernatant was removed. After 10^5^ cells were added to 100 μl of flow cytometry staining buffer, surface staining antibodies against CD45 (Abcam, UK, EP322Y), HLA-DR (Abcam, UK, EPR3692), CD34 (Abcam, UK, EP373Y), CD90 (Abcam, UK, EPR28145-53), and PDGFRA (Abcam, UK, EPR5480) were added for 15 min at room temperature, and the iso control antibody was prepared for staining. Excess antibody was washed off by centrifugation, and flow cytometry staining buffer was added, followed by flow cytometric analysis.

### DNA extraction and next-generation sequencing

We performed targeted panel sequencing in all cell types using a high-depth NGS approach. The DNA was extracted from cells using the Qiagen DNA Extraction Kit (Qiagen, #13,323). Genomic DNA fragments were spliced and modified for sequencing with the NEBNextillinautraII DNA Library Preparation Kit. After library establishment was completed, high-throughput sequencing was performed using the Illumina Nova Seq 6000 platform. The NGS panel had an average sequencing depth of 10,000X and 98% coverage. DNA sequences from the assay samples were compared to the reference sequence hg19 (GRCh37) and analysed to determine the possible variants.

### Adipogenic differentiation of FAPs

Inducing differentiation in FAPs was carried out as per the provided protocol by the manufacturer. In brief, FAPs and their control counterparts were seeded into 6-well plates, maintaining a cell density of 200,000 cells per well. Once confluence reached 90%, the initial medium was switched to an adipogenic differentiation medium A (Cyagen, USA, HUXMD-90,031) and maintained for three days. Subsequently, medium A was substituted with medium B (Cyagen, USA, HUXMD-90,031) for a day. After completing three cycles (totaling 12 days) of this alternating medium A and B regimen, lipid droplet formation was assessed through Oil Red O staining.

### Oil Red O staining

Oil Red O staining was performed according to the instructions of the Modified Oil Red O Staining Kit (Beyotime, C0158S). Quantitative analysis of Oil red O staining area was determined using Image-Pro Plus 6.0 software.

### RNA extraction and quantitative real-time polymerase chain reaction (RT‒qPCR)

Total RNA was isolated from cultured cells utilizing Trizol reagent (Takara, Japan, 9108). cDNA synthesis was performed by following the instructions of PrimeScript™ RT Master Mix (Takara, Japan, RR036A). The TB Green Premix Ex Taq (Takara, Japan, RR820A) facilitated the conduct of RT-qPCR assays on an ABI 7900HT platform, with GAPDH serving as the normalization gene. Primer details are provided in Additional file 2. Table S2. Relative gene expression was evaluated using the 2^−ΔΔct^ approach.

### Western blot assay

Cultured cells were lysed for 30 min with RIPA lysis buffer supplemented with protease inhibitor (Roche, Mannheim, Germany). Protein concentrations were determined with a BCA protein assay kit (Epizyme, China, ZJ102). The proteins were mixed with protein sample loading buffer (Epizyme, China, LT101) at a 4:1 ratio and then treated at 100 °C for 7 min before being transferred to 0.45 μm polyvinylidene difluoride (PVDF) membranes. The membranes were placed and blocked in 5% skim milk solution for 2 h at room temperature and then washed with PBST. The separated proteins were then incubated with primary antibodies: anti-GAPDH (Proteintech, USA, 60004-1-Ig), anti-PIK3CA (CST, USA, 4249T), anti-AKT (CST, USA, 4691 S), anti-p-AKT (CST, USA, 4060T), anti-mTOR (CST, USA, 2983T), anti-p-mTOR (CST, USA, 5536T), anti-FKBP5 (Proteintech, USA, 67874-1-Ig), anti-PPAR γ (CST, USA, 2435 S), anti-C/EBP α (Proteintech, 18311-1-AP), and anti-FABP 4 (Proteintech, 67167-1-Ig) at 4 °C overnight. The membranes were incubated with peroxidase-conjugated secondary antibody at room temperature on the next day. Quantitative analysis was performed on the immunoreactive bands with Image J software.

### Lentivirus transfection

The lentiviral vector containing short hairpin (sh) RNAs against human PIK3CA and FKBP5 and the PIK3CA and FKBP5 overexpression lentivirus were constructed by Zuorun Biotechnology (Shanghai, China, Additional file 3. Table S3). FAPs were placed into 6-well plates (2 × 10^5^ per well) and cultured until the cell density reached 50%. Then, FAPs were infected with virus (MOI = 10) for 10 h with 10 μl of polybrene. Afterwards, the virus-containing medium was replaced with basal medium, and incubation was continued for 48 h. Puromycin was used to remove untransfected cells. The expression of PIK3CA and FKBP5 was analyzed by western blotting and RT‒qPCR.

### SVF isolation and cell viability assay

Each fresh sample underwent SVF extraction. Initially, samples were rinsed multiple times in Hank’s balanced salt solution (14,025,126, Gibco). This was followed by digestion using 0.15% collagenase I (17,100,017, Gibco) enriched with 4% Penicillin-Streptomycin (15,140,122, Gibco) at 37 °C for half an hour. The resultant pellet was suspended in high-glucose Dulbecco’s Modified Eagle’s Medium (10,569,044, Gibco) containing 10% fetal bovine serum (10,099,141, Gibco), passed through a 100-μm mesh, and centrifuged at 4 °C for 5 min. The cell suspensions were then merged with Hank’s balanced salt solution, treated with red blood cell lysis buffer at ambient temperature for 5 min to eliminate erythrocytes, followed by another centrifugation and suspension in Hank’s balanced salt solution mixed with 0.04% bovine serum albumin (A1933-5G, Sigma), and passed through a 40-μm filter. After the final centrifugation, cells were suspended in Dulbecco’s Phosphate Buffered Saline minus Ca^2+^ and Mg^2+^ (14,190,144, Gibco). This single-cell mixture was then combined with an identical volume of AOPI Staining Solution (Logos Biosystems) for incubation. A LUNA-FL Fluorescence Cell Counter (Logos Biosystems) assessed cell concentration and viability, which exceeded 90% for all samples intended for scRNA-seq analysis.

### Primary analysis of raw read data (scRNA-seq)

Gene expression profiles were established from raw reads using CeleScope v1.15.0 (Singleron Biotechnologies), employing the standard settings. In sum, R1 reads were analyzed to retrieve and rectify Barcodes and UMIs. R2 reads underwent trimming to remove adapter sequences and poly A tails, and these trimmed reads were then mapped to the GRCh38 (hg38) genome utilizing STAR (v2.6.1b). Reads that uniquely aligned were allocated to genes via FeatureCounts (v2.0.1). Reads sharing identical cell barcodes, UMIs, and gene identifiers were clustered, forming the basis for the gene expression matrix, which was subjected to subsequent analytical procedures.

### Quality control, dimension-reduction and clustering

Utilizing Scanpy v1.8.1 on Python 3.7, quality control, dimensionality reduction, and clustering were conducted. The expression matrix for each dataset underwent filtering based on several criteria: (1) Removal of cells with fewer than 200 gene counts or those in the top 2% of gene counts; (2) Exclusion of cells within the top 2% of UMI counts; (3) Elimination of cells where mitochondrial content exceeded 15%; (4) Omission of genes present in fewer than 5 cells. Post-filtering, a total of 55,583 cells were preserved for further analysis, averaging 1,353 genes and 3,269 UMIs per cell. Normalization of the raw count matrix was executed per cell total counts followed by a logarithmic transformation, producing a normalized data matrix. By specifying flavor as ‘seurat’, the top 2,000 highly variable genes were identified. Subsequent Principle Component Analysis (PCA) on this subset resulted in the selection of the top 17 principal components for further clustering and dimensional reduction applications. Employing the Louvain algorithm with a resolution parameter set to 1.0 facilitated the division of cells into 22 distinct clusters. Visualization of cell clusters was achieved through Uniform Manifold Approximation and Projection (UMAP).

### Differentially expressed genes (DEGs) analysis

DEGs were pinpointed utilizing the scanpy.tl.rank genes groups function, which employs the Wilcoxon rank sum test under default settings. Genes that were present in over 10% of cells in any compared cell groups and exhibited an average log(Fold Change) above 0.25 were classified as DEGs. The adjusted *p*-value was determined through the Benjamini-Hochberg correction method, with a threshold of 0.05 set to assess statistical significance.

### Pathway enrichment analysis

To explore the functions associated with FAPs, we utilized Gene Ontology (GO) and Kyoto Encyclopedia of Genes and Genomes (KEGG) pathway analyses through the “clusterProfiler” R package, version 3.16.1. Pathways yielding a *p*.adj value below 0.05 were deemed significantly enriched and their corresponding significant pathways were depicted in bar plots. Furthermore, Gene Set Enrichment Analysis (GSEA) was applied to the gene sets of specific clusters. For the Gene Set Variation Analysis (GSVA) on pathway enrichment, we employed the mean gene expression per cell type as the input dataset. We referenced Gene Ontology gene sets across molecular function (MF), biological process (BP), and cellular component (CC) for this analysis.

### Subpopulation-specific regulon analysis

Regulon analysis was conducted using the SCENIC R package to pinpoint key regulators that contribute to cellular diversity across subpopulations. In summary, we identified coexpression modules comprising genes that exhibit coexpression with regulatory elements. We only maintained those modules demonstrating significant enrichment for the motifs of these regulators, which are known as regulons. The activity of each regulon within individual cells was then scored. By evaluating the average scores of regulon activity within a subpopulation, we were able to distinguish regulons that are specific to certain subpopulations.

### Pseudotime trajectory analysis

The differentiation trajectory of FAPs subtypes was mapped using the Monocle2 software, version 2.10.0. To establish this trajectory, we identified the top 2000 variable genes utilizing the FindVariableFeatures function from Seurat, version 3.1.2. Dimensional reduction was then achieved through the DDRTree method. Visualization of the trajectory was accomplished with the plot cell trajectory function within Monocle2.

### Histology and immunohistochemistry

Tissue samples underwent fixation in paraformaldehyde overnight, followed by embedding in paraffin, and slicing. The slices were then subjected to staining procedures using hematoxylin and eosin (H&E). For the purpose of immunohistochemistry, slices were treated with primary antibodies directed against FKBP5 (Proteintech, USA, 67874-1-Ig), and incubated overnight at 4 °C. The subsequent day involved incubation with an HRP-linked secondary antibody, counterstaining with hematoxylin, and development using diaminobenzidine. Quantitative assessments were carried out utilizing Image-Pro Plus 6.0 software.

### Immunofluorescent staining of tissue sections

Sections of tissue were first deparaffinized, followed by antigen retrieval using either EDTA (pH = 9.0) or sodium citrate (pH = 6.0) solutions, applying high pressure for 2 min. Afterward, the sections were incubated with 10% goat serum at ambient temperature for one hour to block non-specific binding, then treated with primary antibodies overnight at 4 °C. The sections underwent three washes with Phosphate Buffered Saline before being incubated with Alexa Fluor 488 goat anti-rabbit (A-11,008, Invitrogen) and Alexa Fluor 594 goat anti-mouse (ab150116, Abcam) secondary antibodies for 40 min at room temperature. Finally, sections were coverslipped using a 4′,6-diamidino-2-phenylindole (DAPI) based mounting medium (ZLI-9557, ZSGB-BIO). The primary antibodies used for immunofluorescent staining included: PDGFRA (Abcam, UK, ab203491), DPP4 (Proteintech, USA, 68383-1-Ig), PRG4 (Abcam, UK, ab28484) and APOE (Proteintech, USA, 18254-1-AP).

### Statistical analysis

Data are expressed as the means ± SDs. The statistical significance of differences was evaluated with one-way analysis of variance (ANOVA) or Student’s t test. *P* values < 0.05 were considered significant. All dates are processed by SPSS 26.0 (IBM, USA) and GraphPad Prism software (version 8). All experiments were repeated at least three times.

## Results

### Clinical characteristic of FIL patients

**Fig. 1:**
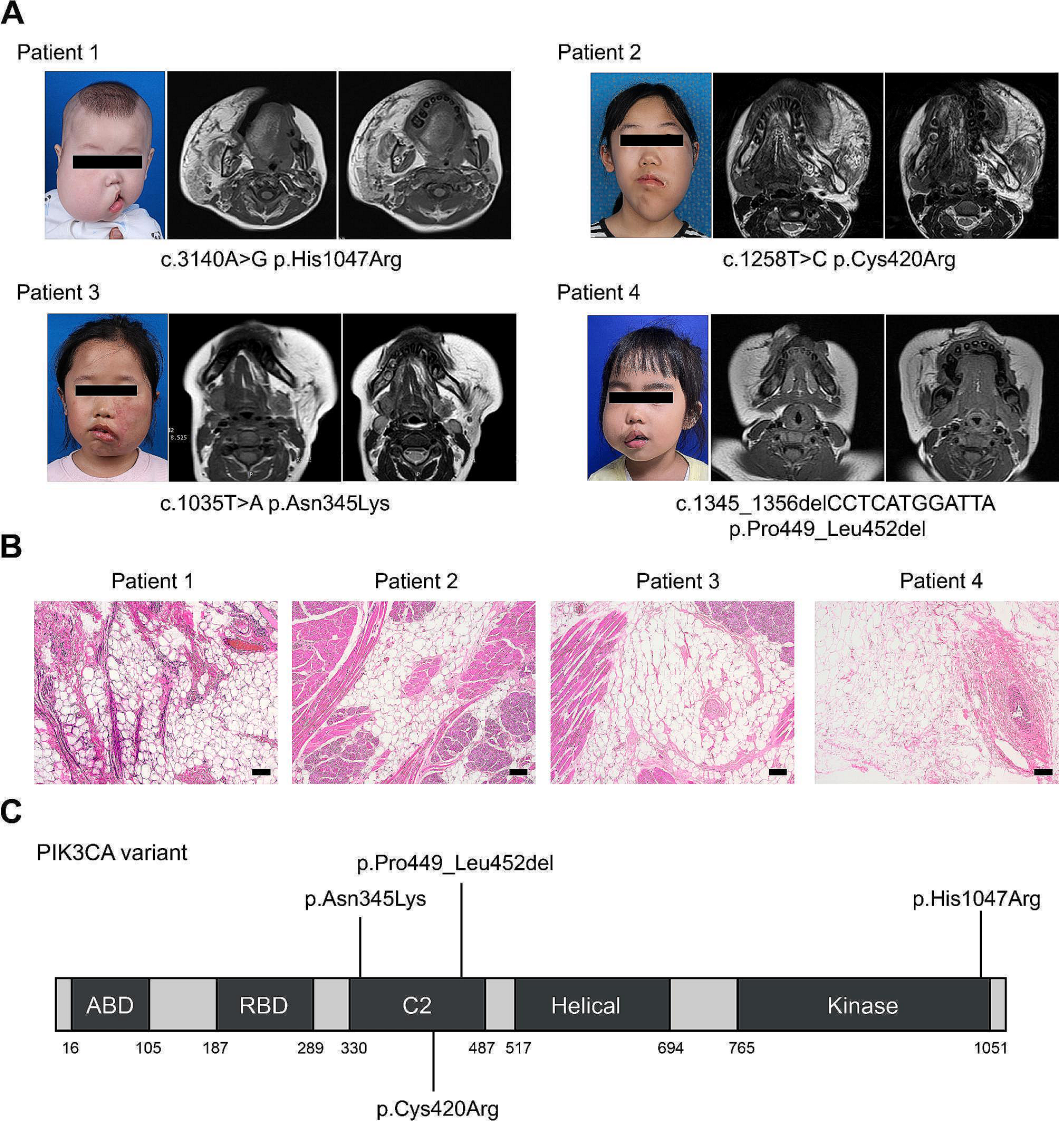
Clinical data of patients with FIL. **A**: Clinical and radiological manifestations of four patients. **B**: Histological characteristics of four patients. **C**: Distribution of PIK3CA variant loci across the p110α domains in the four patients

The clinical information of all FIL patients and controls (CON) included in this study is documented in Table S1. Considering the potential heterogeneity of adipose tissue at different locations [15], we used adipose tissue from patients who underwent surgery for other facial skin lesions as controls. To accurately delineate the cellular heterogeneity of FIL adipose tissue, we isolated the SVF from facial adipose samples of four FIL patients and three control patients and performed scRNA-seq. There was no statistical difference in age or gender between these two groups (Age: *P* = 0.5385; Sex: *P* = 0.714). The clinical and molecular features of these four FIL patients are presented in Fig. 1. All patients exhibited accumulation of the subcutaneous adipose tissue on the face as revealed by magnetic resonance imaging (Fig. 1A), accompanied by infiltration into surrounding muscles or glands (Fig. 1B). We collected adipose tissue samples obtained during surgery and performed next-generation sequencing. Our results revealed that all patients harbored somatic pathogenic variants in PIK3CA, albeit at different loci. Specifically, Patient 1–3 carried the PIK3CA missense variants p.His1047Arg, p.Cys420Arg, and p.Asn345Lys, respectively. Meanwhile, Patient 4 carried a frameshift variant p.Pro449_Leu452del. Three patients had variants in the C2 domain, and one had a variant in the kinase domain (Fig. 1C).

### Single-cell sequencing revealed cellular diversity and heterogeneity in facial adipose tissue

**Fig. 2:**
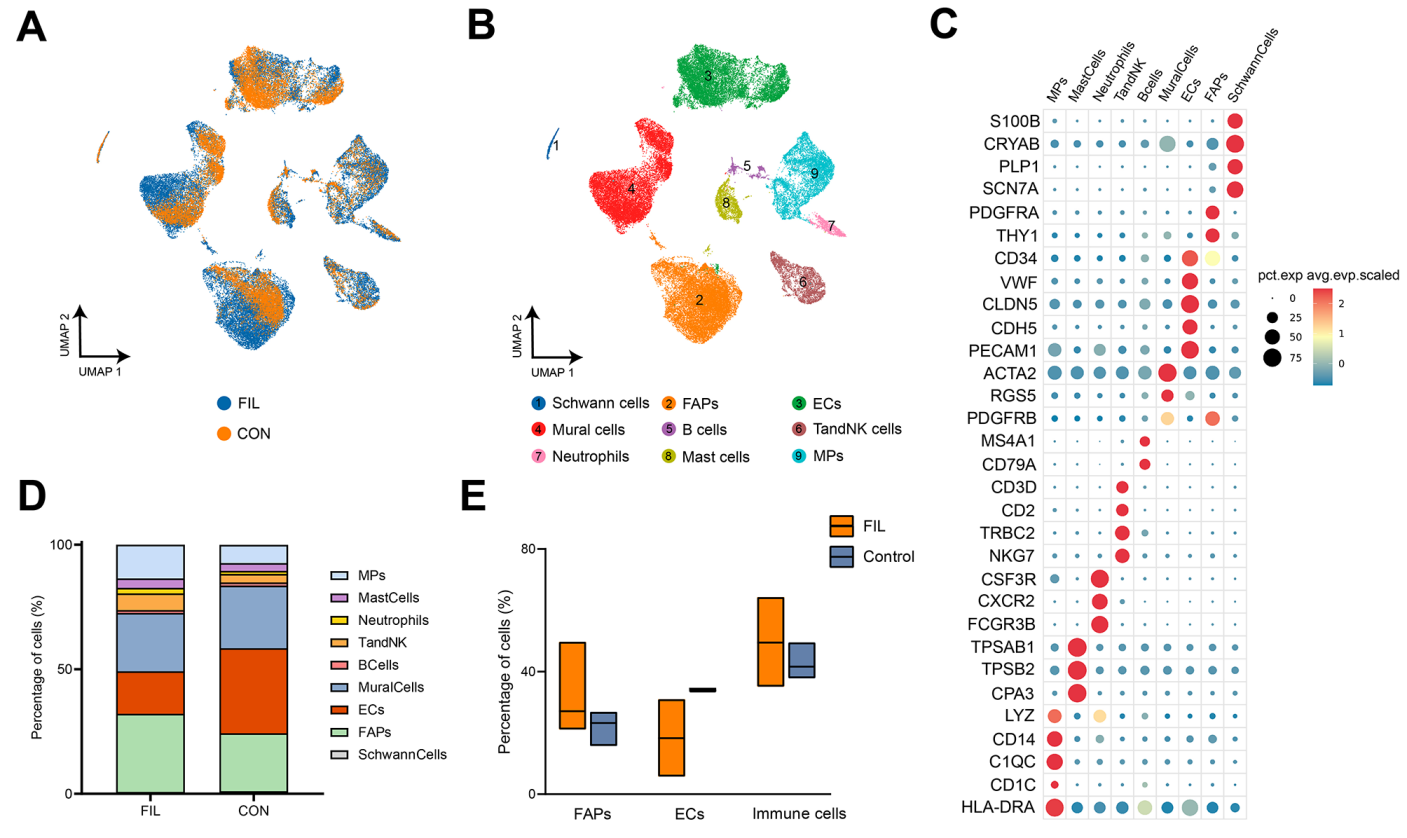
Landscape of cells in the SVF of adipose tissues from patients with or without FIL. **A**: Uniform Manifold Approximation and Projection (UMAP) plot showing the origins of cells. **B**: UMAP plot of single cells showing 9 major cell types by manual annotation. **C**: Bubble heatmap showing expression levels of selected marker genes for each cell type. **D**: Relative proportion of each cell lineages. **E**: Boxplot showing the proportion of major cell types from patients with (*n* = 4) or without (*n* = 3) FIL

Following strict quality control, we collected single-cell transcriptomic data from 55,583 cells (FIL: 35,864, CON: 19,719) for subsequent analysis (Fig. 2A). Initial unbiased clustering identified nine major cell lineages (Fig. 2B), with representative markers for each cell lineage exhibited in Fig. 2C. Among the stromal cells, FAPs were identified by CD34, THY1, and PDGFRA. Vasculature-related lineages included endothelial cells (marked by VWF, CLDN5, and CDH5) and mural cells (marked by ACTA2 and RGS5). Immune cells in adipose tissue could be divided into two major clusters: (i) T and NK clusters, which included T cells, natural killer (NK) cells, and NKT cells, and (ii) mononuclear phagocyte clusters (MPs) composed of monocytes, macrophages, and dendritic cells. Other cell populations such as B cells and mast cells were less prevalent in adipose tissue [16]. Consistently, we also identified a fraction of immune cells, including lymphocytes and myeloid cells, in our specimens. The T and NK cluster exhibited relatively high expression of CD3D, CD2, TRBC2, and NKG7. Other identified lymphocytes included B cells (marked by MS4A1 and CD79A). Myeloid cells included MPs (marked by CD14, C1QC, and HLA-DRA), neutrophils (marked by CSF3R, CXCR2, and FCGR3B), and mast cells (marked by TPSAB1, TPSB2, and CPA3). We also identified a small number of Schwann cells, primarily marked by S100B, PLP1, SCN7A (Fig. 2C).

Cell populations that exhibit significant changes in relative proportions may be associated with disease status. Indeed, we observed certain disparities between FIL and CON in the distribution ratios of different cell populations (Fig. 2D). Notably, the proportion of FAPs was higher in FIL (ranging from 21.1 to 49.7%) compared to CON (ranging from 15.7 to 26.9%), suggesting an increased reserve of FAPs in FIL. Additionally, FIL exhibited more immune cell infiltration (ranging from 35.1 to 64.3% in FIL versus 37.8–49.5% in CON). However, the presence of endothelial cells in FIL was decreased (ranging from 5.77 to 31.01% in FIL versus 33.21–34.42% in CON) (Fig. 2E). Considering that FAPs are primarily involved in the generation of adipocytes and the accumulation of lipid droplets, processes that are most relevant to the clinical phenotype, we focused our analysis on the FAPs population.

### Distinct FAPs cluster identified in FIL and CON

We performed a detailed analysis of the FAPs in the FIL and CON groups. As previously reported [17], the FAPs lineage, also known as adipose stem and progenitor cells (ASPC), can be further subdivided into three populations: adipose stem cells (ASCs), which exhibit a stem-like properties and multidirectional differentiation potential; the preadipocyte population (PreAs), characterized by committed adipogenic function; and the adipogenesis regulators (Aregs) population that can inhibit adipogenic differentiation of other precursor cells [18]. Based on this framework, we sought to analyze the likely functional states of the annotated cell clusters in our samples.

**Fig. 3:**
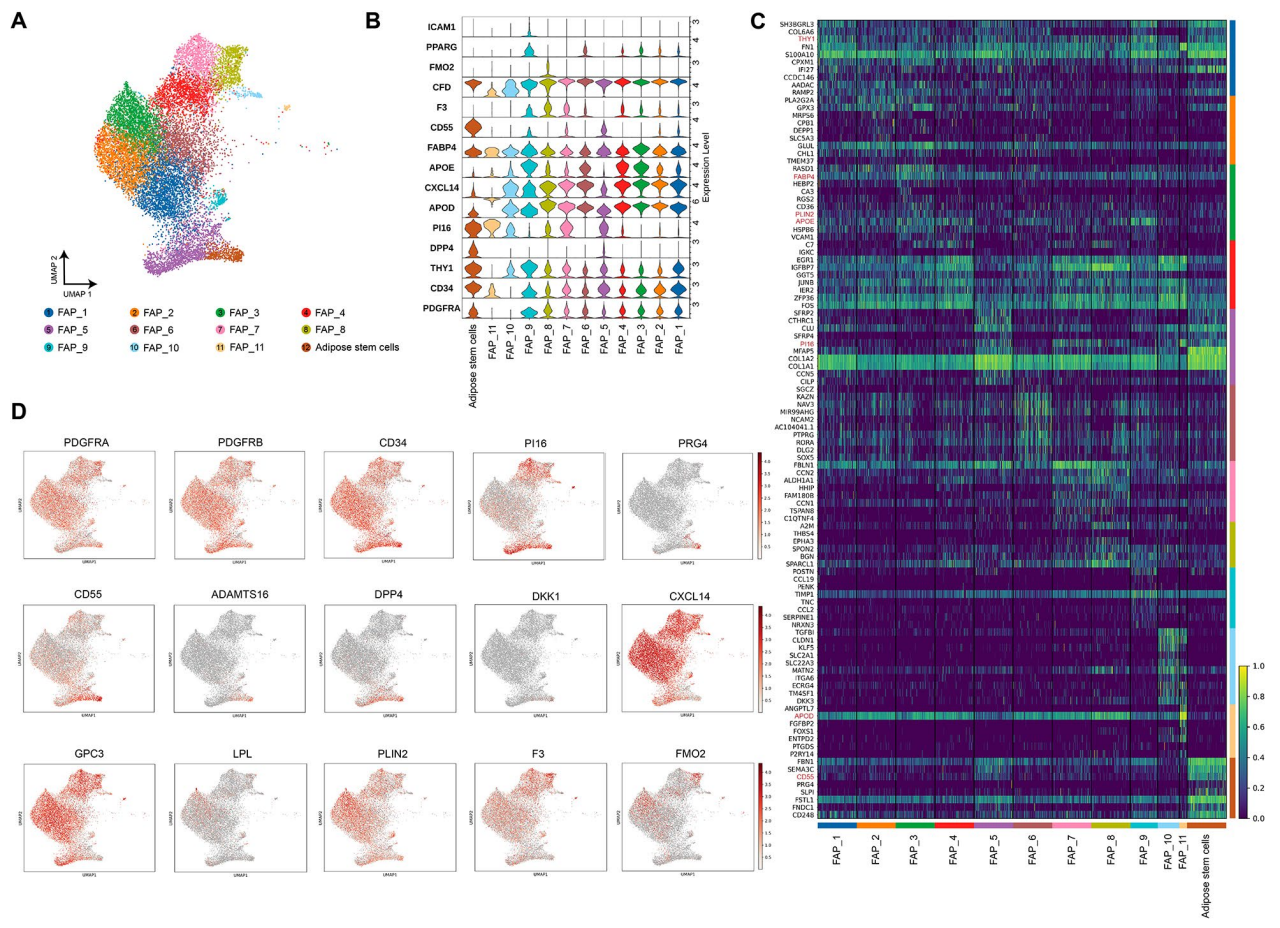
Landscape of FAPs subpopulations. **A**: UMAP visualization of inferred FAPs from adipose tissue identified twelve FAPs subpopulations. **B**: Violin plot showing the expression of marker genes within each cell subpopulations. **C**: Heat map (blue-to-yellow) of scaled expression of marker genes for each cell type and top ten differentially expressed genes (DEGs) for each indicated cell cluster. Genes shown in red were some marker genes used for manual annotations. **D**: Distribution map of the selected genes expressed in different subgroups. The color depth represents the intensity of expression in the samples

We identified 12 subpopulations (Fig. 3A), with all clusters, except for clusters 10 and 11, being recognized by PDGFRA, CD34, or THY1 (Fig. 3B). Clusters 10 and 11 belong to the previously mentioned FAPs population that does not express PDGFRA [16]. Cluster 12 exhibited high expression of the typical ASCs markers: DPP4, CD55, and Pi16 [19]. In addition, specific ASCs markers such as ADAMTS16, DKK1, and PRG4 were uniquely expressed in this group (Fig. 3C, D, and Additional file 4. Figure S1A) [20]. Consequently, it was annotated as adipose stem cells, representing the most pluripotent stem cell population. Cluster 5 exhibited relatively high expression of DPP4 and CD55, but lacked other specific ASC markers, suggesting a partial loss of stemness, yet they still represent ASCs. Cluster 8 expressed high levels of FMO2 and F3 (CD142), which are characteristic markers of Aregs cells [18]. Other clusters were annotated as PreAs, yet they were at different stages of differentiation. Clusters 1, 2, 3, 4, 6, and 9 all had elevated expression of APOE and PPARG, suggesting that they might be in the later stages of adipogenic differentiation [17]. Of these, clusters 3 and 9 had the highest PLIN2 expression, indicating a higher commitment towards mature adipocytes (Fig. 3C, D, and Additional file 4. Figure S1A) [17]. LPL, another adipocyte-specific marker, was rarely observed in distinct cell clusters, possibly due to the exclusion of adipocytes from the SVF. Clusters 7, 10, and 11 comprised earlier-stage PreAs, marked by relatively high GPC3 and APOD expression. Notably, clusters 7 and 11 had elevated PI16 levels, suggesting they might have just initiated adipogenesis (Additional file 4. Figure S1A) [16]. Immunofluorescence confirmed the presence of ASCs and PreAs (Additional file 5. Figure S2A-C). Moreover, we compared the expression of PIK3CA across the various cell clusters. We noted that the FAP cell groups from the FIL cohort (FIL-FAPs) displayed higher levels of PIK3CA expression compared to the control group (Additional file 4. Figure S1B).

**Fig. 4:**
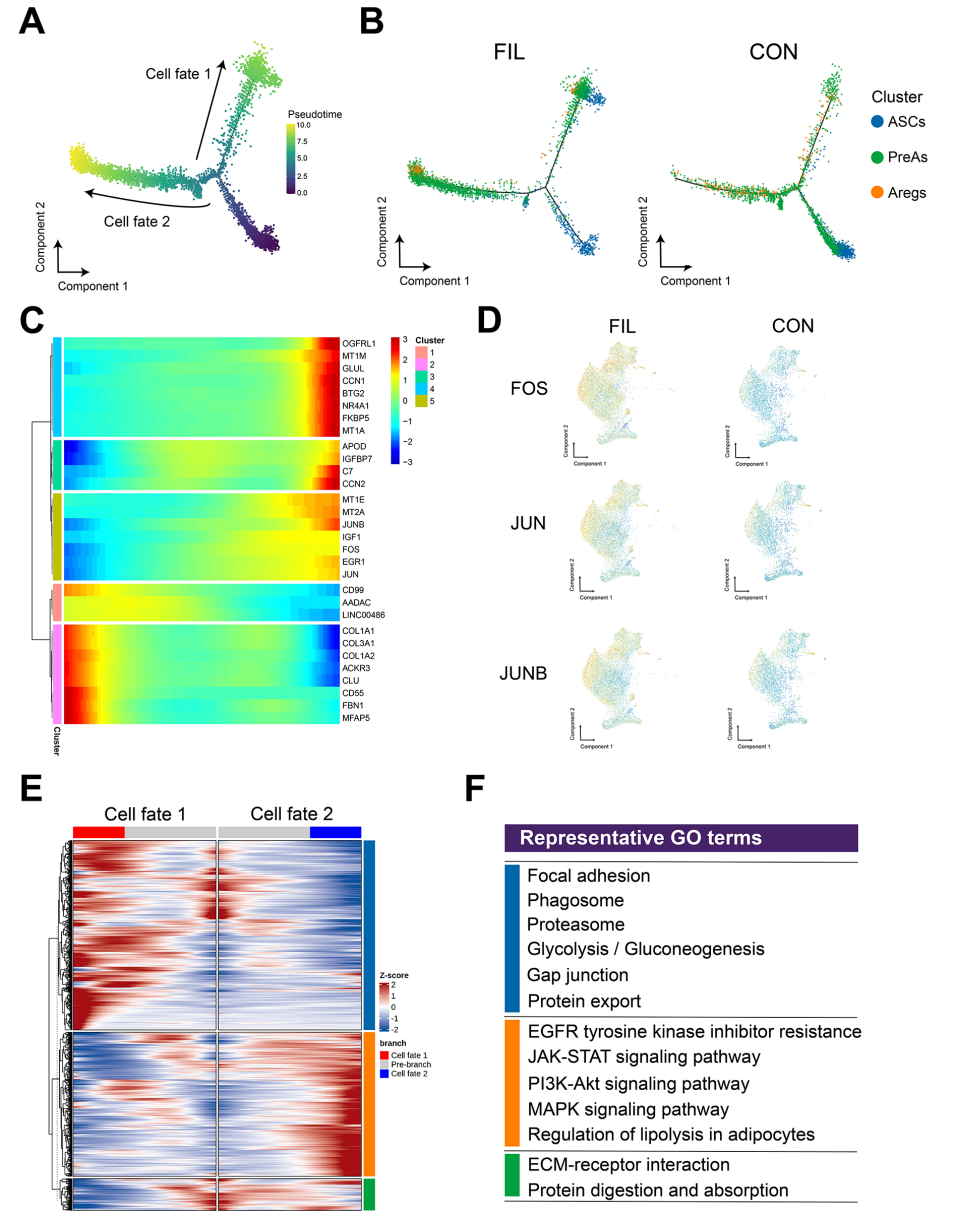
Landscape of FAPs subpopulations. **A**-**B**: Pseudo-time trajectory map of FAPs in FIL and CON classified by manual annotation. **C**: Heatmap showing the dynamic changes in gene expression along the pseudo-time. **D**: Regulon scores of some transcriptional factors in FIL and CON. **E**: BEAM heat map depicting the expression of the branch-dependent genes over pseudotime. Genes are clustered to three modules based on expression patterns across pseudotime. The branch point shown in the middle of heat map is the beginning of pseudotime. Both sides of heat map are the ends of pseudotime. Color bar indicates the relative expression level. Cell fate 1 matches the upper branch and Cell fate 2 matches the lower branch as shown in Fig. 4a. F: Representative GO: BP terms of each module. Adjusted *p* value < 0.05 was considered statistically significant for GO enrichment analysis

We next performed a pseudotime analysis to identify the cell differentiation trajectory. Due to the significant batch effect of the FIL-4 sample (data not shown), we excluded it from the pseudotime analysis. The cell trajectory analysis revealed two distinct cell fates of FAPs (Fig. 4A), with ASPCs from the FIL cohort demonstrating a stronger inclination towards cell fate 2 (Fig. 4B). As pseudotime progressed, we observed a significant upregulation of pro-adipogenic regulators such as members of the activator protein-1 (AP-1) family of transcriptional factors FOS, JUN, and JUNB (Fig. 4C). Moreover, FOS, JUN, and JUNB exhibited elevated regulon scores in FIL-FAPs, suggesting an increased activity under pathological conditions. This likely led to the activation of a broader range of target gene expressions (Fig. 4D). We compared the genes differentially enriched in the two cell fates. Genes enriched in cell fate 1 are involved in focal adhesion and glycolysis/gluconeogenesis. In contrast, cell fate 2 is enriched in various PIK3CA-related signaling pathways such as the JAK-STAT, MAPK, and PI3K-AKT pathways, all of which are implicated in cell proliferation and the promotion of adipogenic differentiation [21, 22]. This might be one of the significant factors contributing to the development of the FIL phenotype. Moreover, cells in fate 2 showed enrichment in the regulation of lipolysis in adipocytes (Fig. 4E, F), suggesting that FIL-FAPs might also exhibit notable alterations in lipid metabolism, which warranted further investigation.

### Heterogeneity of FAPs between the FIL and CON

**Fig. 5:**
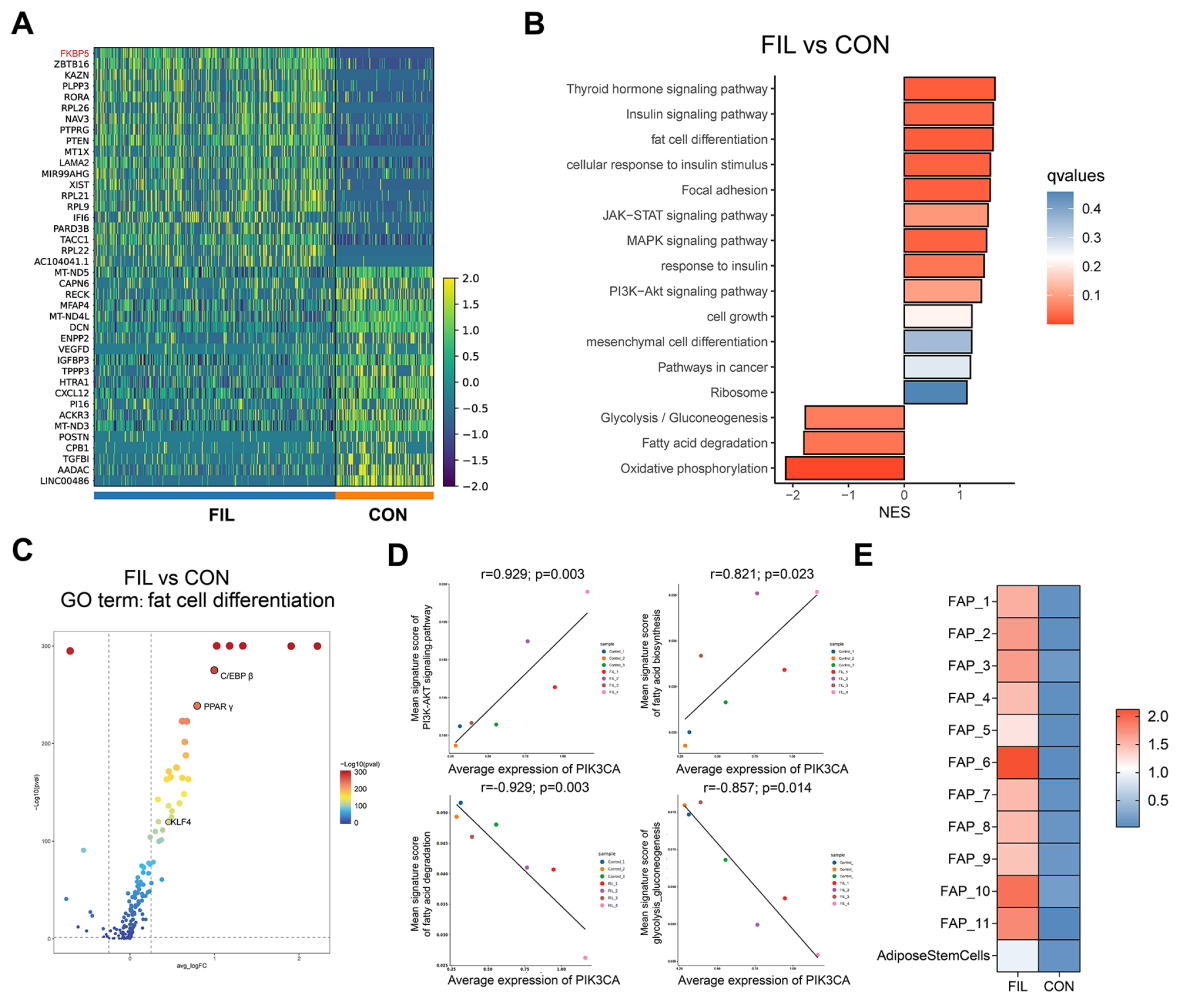
Major DEG in FIL-FAPs and CON-FAPs. **A**: Heat map showing the scaled expression of top 20 marker genes in FIL-FAPs and CON-FAPs. Vital genes associated with adipogenesis are highlighted with red color. **B**: Bar plot showing significantly enriched KEGG pathways in DEGs between FIL-FAPs and CON-FAPs. The color saturation of bars indicating the significance of enrichment. **C**: Volcano plot showing the differences in the expression of genes involved in fat cell differentiation in FIL-FAPs and CON-FAPs. **D**: Correlation analysis of PIK3CA expression and related pathways. **E**: Heat map showing the expression of FKBP5 in the FAP subgroups of FIL and CON

We further compared genes distinctly expressed between the two groups of FAPs. Notably, we found that certain gene associated with adipogenesis, such as FKBP5, was significantly up-regulated in FIL-FAPs (Fig. 5A, Additional file 6. Table S4) [23, 24]. Further gene set enrichment analysis (GSEA) indicated that compared to the CON-FAPs, FIL-FAPs were enriched in fat cell differentiation, mesenchymal cell differentiation, and cell growth pathways (Fig. 5B). We also observed relatively elevated expression of pivotal genes involved in the regulation of adipocyte differentiation, including PPAR γ, KLF 4, and CEBP β (Fig. 5C) [25]. This suggested an enhanced adipogenic capacity in FIL-FAPs, thereby potentially promoting the development of lipomatosis. Furthermore, the FIL-FAPs displayed enrichment in numerous hormonal signaling pathways, indicating differential hormonal responsiveness when compared to the CON-FAPs. Interestingly, we noted a downregulation in the fatty acid degradation capability in FIL-FAPs, which may facilitate lipid accumulation (Fig. 5B). Subsequent correlation analysis revealed a significant correlation between PIK3CA expression and the PI3K-AKT pathway as well as the fatty acid synthesis pathway. Conversely, a negative correlation was observed with both the glycolysis pathway and fatty acid degradation (Fig. 5D). These findings suggested that PIK3CA variants may be an intrinsic factor underlying these phenotypes.

### FKBP5 was regulated by PI3K-AKT pathway

**Fig. 6:**
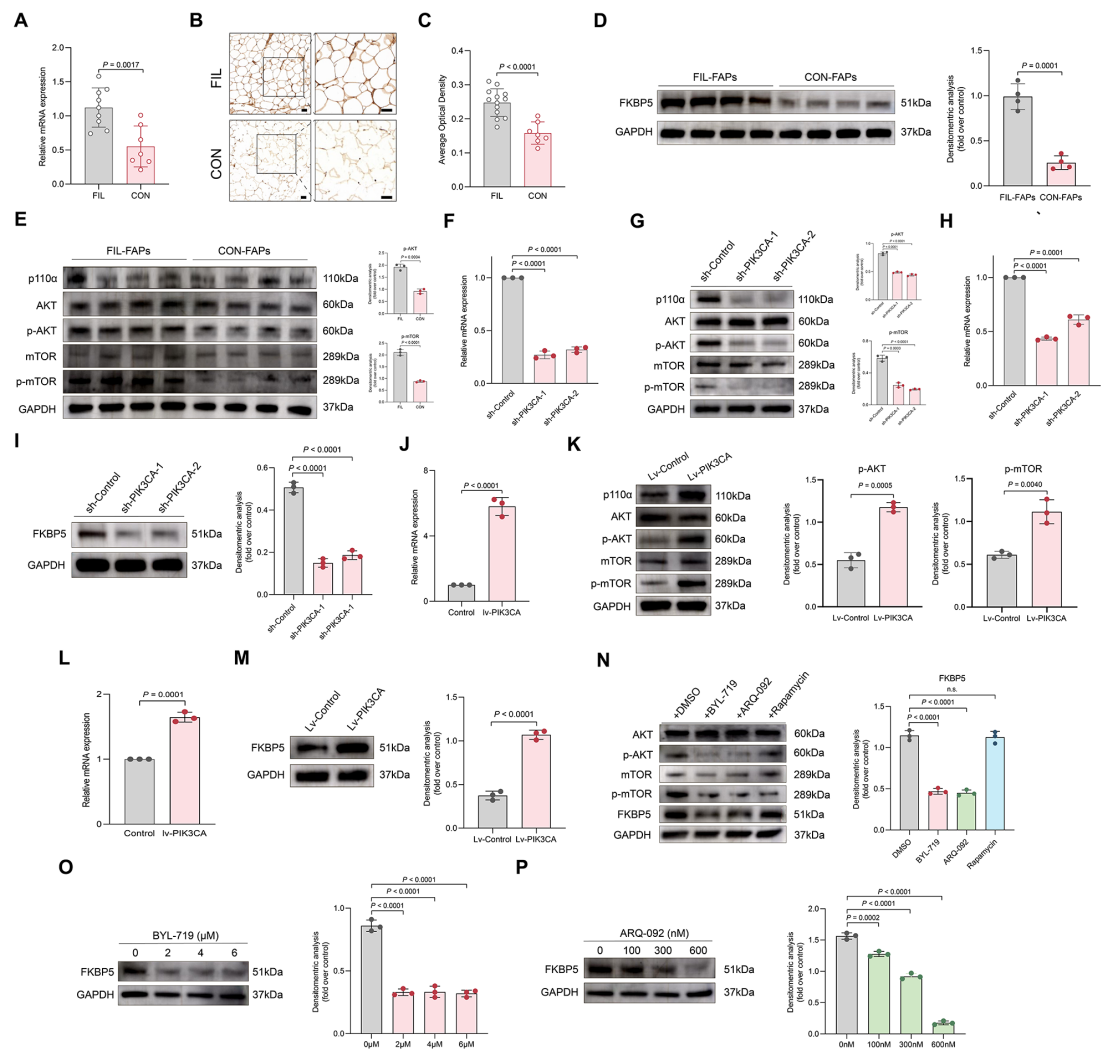
Expression of FKBP5 was regulated by PI3K-AKT pathway. **A**: The mRNA level of FKBP5 in FIL and normal adipose tissues. **B**: FKBP5 expression levels were visualized by immunohistochemical staining in FIL and normal adipose tissues. Scale bar: 100 μm. **C**: Statistical results of FKBP5 expression levels in FIL and normal adipose tissues. **D**: Western blot analysis of the expression of FKBP5 in FIL-FAPs and CON-FAPs (*n* = 4). **E**: The expression of proteins downstream of PI3K/AKT pathway were detected in FIL-FAPs and CON-FAPs by Western blot (*n* = 4). **F**: RT-qPCR verified the silencing of PIK3CA after transfection of the shRNA into FIL-FAPs (*n* = 3). **G**: The levels of proteins downstream of PI3K/AKT pathway in PIK3CA-knockdown FIL-FAPs were determined by western blotting (*n* = 3). Data are presented as the mean ± SD. **H**-**I**: RT-qPCR (**H**) and Western blot (**I**) showed that the expression of FKBP5 was decreased after shRNAs transfection in FIL-FAPs (*n* = 3). **J**: RT-qPCR verified the overexpression of PIK3CA after transfection of the lentivirus into CON-FAPs (*n* = 3). **K**: Western blot showed that PIK3CA was overexpressed in CON-FAPs after transfected with lentivirus carrying PIK3CA gene (*n* = 3). **L**-**M**: RT-qPCR (**L**) and Western blot (**M**) showed that the expression of FKBP5 was increased after overexpression of PIK3CA in CON-FAPs (*n* = 3). **N**: The protein levels of PI3K/AKT and FKBP5 in FIL-FAPs pretreated with BYL-719, ARQ-092, and rapamycin for 24 h (*n* = 3). **O**: The expression levels of FKBP5 were determined by western blotting in FIL-FAPs after BYL-719 treatment for 24 h (*n* = 3). P: The expression levels of FKBP5 were determined by western blotting in FIL-FAPs after ARQ-092 treatment for 24 h (*n* = 3). Experiments were independently replicated at least three times with similar results (biological replicates). Data were analyzed by unpaired two-sided Student’s t tests (D, K, M) or one-way ANOVA (F, G, I, N, O, P), and are presented as mean ± SD with three replicate experiments. Scale bar: 50 μm. Full-length blots are presented in Additional file 10. Figure S6

Given that FKBP5 is the top-ranked gene showing differential expression between the two groups (Fig. 5A), and its consistently higher expression in FIL-FAPs across all differentiation stages (Fig. 5E), we decided to explore its role in FIL-FAPs. We first assessed FKBP5 expression in adipose tissue. RT-qPCR revealed an elevated FKBP5 level in the adipose tissue derived from FIL (Fig. 6A). Immunohistochemistry on 13 samples of FIL adipose tissue and 7 samples of normal facial adipose tissue revealed an enhanced overall enhanced presence of FKBP5 in FIL adipose tissue (Fig. 6B, C). We isolated FIL-FAPs from FIL and CON-FAPs from normal adipose tissue, both expressing the identical FAPs markers (Additional file 7. Figure S3). Consistently, western blotting showed a significant increase in FKBP5 level in FIL-FAPs as compared to the CON-FAPs (Fig. 6D). Next-generation sequencing of FIL-FAPs revealed that all specimens harbored PIK3CA variants (Additional file 8. Figure S4). The activation degree of the PI3K-AKT pathway in FIL-FAPs was higher than that in the CON-FAPs (Fig. 6E), implying that the elevated expression of FKBP5 might be associated with the hyperactivated PI3K-AKT pathway. It was noteworthy that the expression levels of PIK3CA protein in the two groups of FAPs did not show a significant difference. This observation was consistent with the functional role of PIK3CA variants, as the variants in PIK3CA often led to the relief of inhibition by p85α. Consequently, this allowed for sustained activation and promoted the phosphorylation of downstream proteins [26].

To verify if FKBP5 was regulated by PI3K-AKT pathway and the effect of this pathway in adipogenesis, we utilized two distinct short hairpin RNAs (sh-PIK3CA-1 and sh-PIK3CA-2) to silence PIK3CA. Following successful silencing of PIK3CA (Fig. 6F), we observed a significant decrease in the phosphorylation levels of AKT and mTOR in FIL-FAPs (Fig. 6G). Knocking down PIK3CA led to a reduced adipogenesis capacity of FIL-FAPs in vitro, as evidenced by a decrease in lipid droplet accumulation and a reduction in the expression of adipogenic markers (Additional file 9. Figure S5A-C). Furthermore, the adipogenic capacity of FIL-FAPs in vivo was also impaired following the knockdown of PIK3CA (Additional file 9. Figure S5D-F). Significantly, silencing PIK3CA led to a notable reduction in FKBP5 expression (Fig. 6H, I). We further observed whether overexpression of PIK3CA would affect the expression of FKBP5. CON-FAPs, upon reaching 60–70% confluence, were transfected with either a lentiviral vector harboring the human PIK3CA gene (lv-PIK3CA) or an empty control lentivirus (lv-Control). To assess overexpression efficiency, we conducted RT-qPCR and western blotting, revealing a significant increase in PIK3CA expression and activation level of PI3K-AKT pathway in the lv-PIK3CA group (Fig. 6J, K). PIK3CA overexpression promoted adipogenesis in CON-FAPs in vitro (Additional file 9. Figure S5G-I) and in vivo (Additional file 9. Figure S5J-L). Following the overexpression of PIK3CA, we observed an upregulation in both the mRNA and protein levels of FKBP5 (Fig. 6L, M). We next treated FAPs with BYL-719 (a PI3K inhibitor), ARQ-092 (an AKT inhibitor), and rapamycin (an mTOR inhibitor). Notably, treatment with BYL-719 and ARQ-092 led to a decrease in FKBP5 levels, while rapamycin had no significant impact on its expression (Fig. 6N). This suggested that the regulation of FKBP5 expression may be mediated by the PI3K-AKT pathway but not through mTOR. To confirm this, we treated FAPs with varying concentrations of BYL-719 and ARQ-092. We observed that both inhibitors exhibited a dose-dependent suppressive effect on FKBP5 (Fig. 6O, P). Taken together, these findings indicated that FKBP5 expression was regulated by the PI3K-AKT pathway, and the overactivation of the PI3K-AKT pathway due to PIK3CA variants led to elevated FKBP5 expression in FIL-FAPs.

### FKBP5 promoted adipogenesis of FAPs

**Fig. 7:**
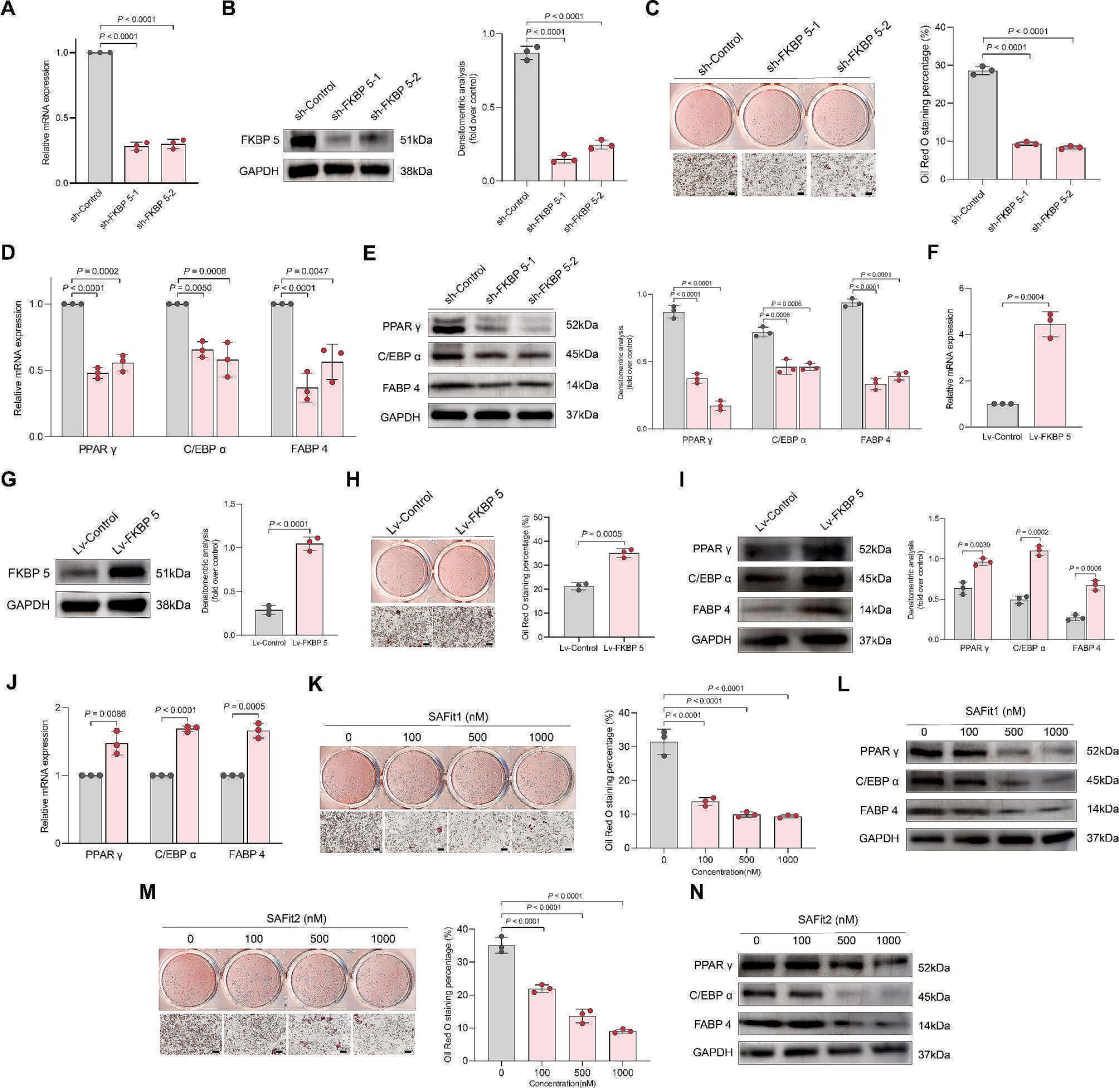
FKBP5 is crucial for adipogenesis of FAPs in vitro. A-B: RT-qPCR (**A**) and Western blot (**B**) showed that FKBP5 was silenced after shRNAs transfection in FIL-FAP (*n* = 3). **C**: Oil Red O staining of sh-Control, shFKBP5-1 and shFKBP5-2 were conducted after adipogenic induction for 12 days. Scale bar: 100 μm. **D**: The mRNA levels of PPAR γ, C/EBP α, and FABP4 in FKBP5-knockdown FIL-FAPs were measured by RT-qPCR on Day 3 of adipogenic differentiation. Data shown here are mean ± SD from three independent experiments. **E**: The protein levels of PPAR γ, C/EBP α, and FABP4 in FKBP5-knockdown FIL-FAPs were measured by Western blot on Day 3 of adipogenic differentiation (*n* = 3). **F**-**G**: RT-qPCR (**F**) and Western blot (**G**) showed that FKBP5 was overexpressed in CON-FAPs after transfected with lentivirus carrying FKBP5 gene (*n* = 3). **H**: Oil Red O staining of lv-Control and lv-FKBP5 were conducted after adipogenic induction for 12 days. Scale bar: 100 μm. **I**: The protein levels of PPAR γ, C/EBP α, and FABP4 in FKBP5-overexpressed CON-FAPs were measured by Western blot on Day 3 of adipogenic differentiation (*n* = 3). **J**: The mRNA levels of PPAR γ, C/EBP α, and FABP4 in FKBP5-overexpressed CON-FAPs were measured by RT-qPCR on Day 3 of adipogenic differentiation (*n* = 3). **K**: The mature adipocytes with lipid droplets were visualized by Oil Red O staining on day 12 after SAFit1 treatment. **L**: The expression of adipogenic marker genes PPAR γ, C/EBP α, and FABP4 was determined by Western blot in SAFit1 treated FIL-FAPs on day 3 of adipogenic differentiation (*n* = 3). **M**: The mature adipocytes with lipid droplets were visualized by Oil Red O staining on day 12 after SAFit2 treatment. **N**: The expression of adipogenic marker genes PPAR γ, C/EBP α, and FABP4 was determined by Western blot in SAFit2 treated FIL-FAPs on day 3 of adipogenic differentiation (*n* = 3). Data were analyzed by unpaired two-sided Student’s t tests (G, I) or one-way ANOVA (B, E, L, N), and are presented as mean ± SD with three replicate experiments (biological replicates). Scale bar: 50 μm. Full-length blots are presented in Additional file 10. Figure S6

Previous research has found that FKBP5 expression in 3T3-L1 preadipocytes progressively increased as adipogenesis advanced, and rapidly relocated from mitochondria to the nucleolus at the onset of adipogenic differentiation [27]. To verify whether FKBP5 is involved in the aberrant adipogenesis in FIL, we first knocked down FKBP5 in FIL-FAPs using lentivirus (sh-FKBP5-1, sh-FKBP5-2). RT-qPCR confirmed significant downregulation of FKBP5 mRNA expression following knockdown (Fig. 7A), corroborated by a western blot assay indicating decreased FKBP5 protein levels (Fig. 7B). During the subsequent adipogenic induction, we observed a significant reduction in lipid droplet accumulation in FKBP5-knocked down FIL-FAPs (Fig. 7C). Consistently, the expression of the key adipogenic transcription regulators PPAR γ, C/EBP α, and FABP 4 was reduced at both mRNA and protein levels following FKBP5 depletion (Fig. 7D, E). We further overexpressed FKBP5 (lv-FKBP5) in CON-FAPs, and validated this using RT-qPCR and immunoblotting (Fig. 7F, G). Overexpression of FKBP5 enhanced lipid accumulation in CON-FAPs during adipogenesis (Fig. 7H), and the expression of major adipogenic markers also increased compared to the control group (Fig. 7I, J). To explore the effect of FKBP5 inhibition, we treated FIL-FAPs with the FKBP5 inhibitor SAFit1. SAFit1 impaired the adipogenic differentiation of FIL-FAPs (Fig. 7K) and reduced the expression of PPAR γ, C/EBP α, and FABP 4 (Fig. 7L). Additionally, we utilized another inhibitor, SAFit2. Additionally, treatment with another FKBP5 inhibitor, SAFit2, demonstrated consistent effects on adipogenesis in FIL-FAPs (Fig. 7M), resulting a dose-dependent reduction in the expression of PPARγ, C/EBPα, and FABP4 (Fig. 7N).

### FKBP5 affects the adipogenesis of FIL-FAPs in vivo

**Fig. 8:**
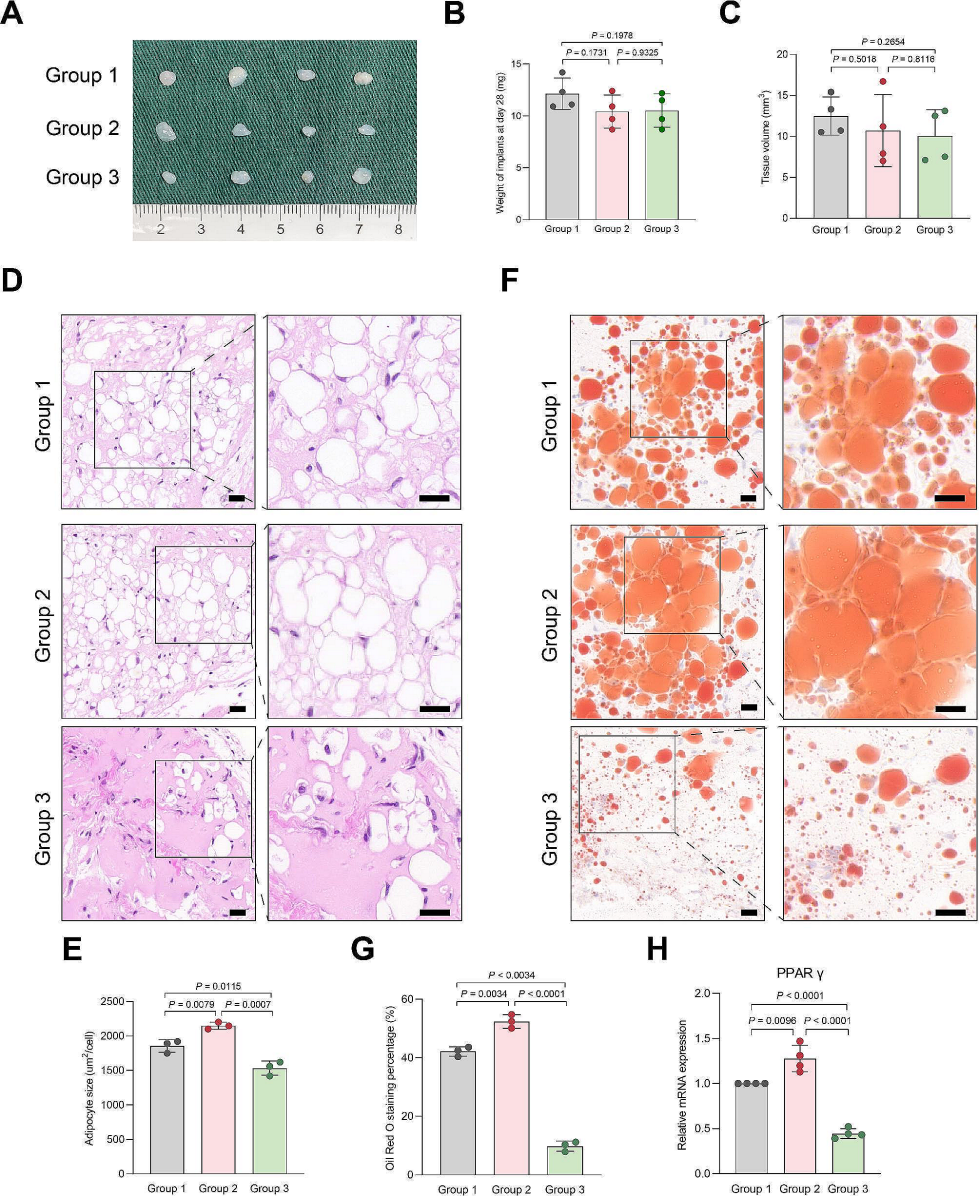
FKBP5 is essential for adipogenesis of FIL-FAPs in vivo. **A**: The macroscopic appearance of Matrigel implants harvested from mice of three groups. **B**-**C**: Statistical analysis of implant volume and weight. **D**-**E**: H&E staining of Matrigel implants collected on day 28 and quantification of adipocytes size. **F**-**G**: Oil Red O staining of Matrigel implants from three groups and quantification of Oil red O staining area. **H**: mRNA level of PPAR γ in three groups. Experiments were independently replicated at least three times with similar results (*n* = 3). Data were analyzed by one-way ANOVA (E, G, H), and are presented as mean ± SD with three replicate experiments (H) or three biological replicates (E, G). Scale bar: 50 μm

To assess the impact of FKBP5 on adipogenesis in vivo, we established a lipoma model in female nonobese diabetic/severe combined immunodeficiency mice by co-implanting FAPs in Matrigel. We designated three experimental groups: FIL-FAPs transfected with empty virus (Group 1); FIL-FAPs transfected with lv-FKBP5 (Group 2); FIL-FAPs transfected with sh-FKBP5 (Group 3). After four weeks, the FAPs-derived xenografts were harvested and analyzed by H&E and Oil Red O staining (Fig. 8A). There was no significant difference in the weight and volume of the grafts among the three groups (Fig. 8B, C). H&E staining revealed that adipocytes in Groups 3 were smaller in size and fewer in number compared to those in Groups 1 and 2 (Fig. 8D, E). Compared to Group 1, the xenografts from Group 3 presented with less Oil Red O positive staining areas, while the Oil Red O staining area in Group 2 was increased (Fig. 8F, G). the mRNA expression levels of PPAR γ were notably lower in Group 3 than in Groups 1 and 2 (Fig. 8H). Collectively, these data suggested that FKBP5 promoted adipogenesis of FIL-FAPs and may serve as a potential therapeutic target for managing excessive adipose accumulation in FIL.

## Discussion

FIL is characterized by excess adipose deposition in the affected facial region. While previous studies have identified PIK3CA somatic variants in adipose tissue of FIL, the downstream effects of these variants within FIL remained largely unexplored. In this study, we conducted the first analysis of the cellular landscape in the non-adipocyte fraction of facial adipose tissues from patients with or without FIL, with a focused investigation on FAPs. All FIL-FAPs were found to carry PIK3CA variants, exhibiting both enrichment and hyperactivation of the PI3K-AKT pathway. We observed a significant upregulation of FKBP5 expression in FIL-FAPs and discovered that its expression was regulated by the PI3K-AKT pathway. Diminishing or inhibiting FKBP5 markedly impaired the adipogenic differentiation of FIL-FAPs, suggesting the potential of FKBP5 as a therapeutic target for FIL.

FIL is categorized as a disease within the PIK3CA-related overgrowth spectrum (PROS). In the context of PROS, nearly a hundred different PIK3CA variant sites have been identified [28]. However, the number of variant loci specifically associated with FIL is comparatively limited. Our previous work compiled PIK3CA variant loci related to FIL reported both at our center and in the literature. This effort revealed that only 10 distinct PIK3CA variant sites have been identified in FIL [2]. In this study, we have identified PIK3CA p.Asn345Lys and p.Pro449_Leu452del in FIL patients for the first time. Although PIK3CA variants at different sites might not uniformly activate the PI3K-AKT pathway to the same extent, they all result in alterations in the expression of downstream proteins [29]. Given the relatively recent introduction of the PROS, there has been limited research exploring the changes in downstream genes within the diseases it encompasses. However, understanding these genetic alterations is crucial for identifying potential therapeutic targets.

FKBP5 has increasingly been identified as a crucial mediator in various signaling cascades, including the PI3K-AKT pathway. A primary mechanism through which FKBP5 impacts the PI3K-AKT pathway is through its interaction with AKT itself. The phosphorylation at the Thr308 and Ser473 residues is essential for the full activation of AKT [30]. In tumor cells, FKBP5 has been demonstrated to bind AKT, leading to the dephosphorylation of AKT at Ser473 without affecting the phosphorylation site at Thr308. Nonetheless, it remains unclear whether FKBP5 is also regulated by the PI3K-AKT pathway in FAPs. To explore this, we manipulated the expression of PIK3CA to alter the activation level of the PI3K-AKT pathway. We observed that increased activation of the PI3K-AKT pathway resulted in elevated FKBP5 expression, whereas decreased activation led to reduced FKBP5 expression. Furthermore, we treated FIL-FAPs with inhibitors of PI3K and AKT and found a dose-dependent decrease in FKBP5 expression. Combined with previous literature, these data suggest a reciprocal regulatory relationship between FKBP5 and the PI3K-AKT pathway.

FKBP5 is known for its involvement in regulating glucocorticoid receptor sensitivity and the stress response [31]. However, recent studies have illuminated its unexpected role in adipogenesis [32]. Adipogenesis is a meticulously regulated process 1. Preadipocytes undergo a complex series of morphological and biochemical changes to become mature adipocytes, which are capable of storing lipids. This differentiation process is primarily regulated by a network of transcription factors, including PPARγ, C/EBP α, and FABP 4, which are pivotal in initiating and driving the adipogenic program [33]. Evidence indicates that FKBP5 expression is upregulated during the early stages of adipocyte differentiation, suggesting its supportive role in this process [27]. The mechanism appears to involve the modulation of PPAR γ, the master regulator of adipogenesis, and other transcription factors critical to this pathway [34]. We discovered that knocking out FKBP5 or inhibiting its function resulted in decreased generation of PPAR γ and affected adipogenic differentiation in FAPs. Conversely, overexpression of FKBP5 promoted the expression of PPAR γ and enhanced adipogenic differentiation, leading to increased lipid droplet accumulation. This outcome aligns with findings from previous studies, reinforcing the pivotal role of FKBP5 in the regulation of adipogenesis [35].

Understanding the role of FKBP5 in adipogenesis provides valuable insights into the pathophysiology of adipose overgrowth. This is not limited to FIL but can be extended to other diseases associated with somatic PIK3CA variants and excessive adipose tissue growth. Elucidating FKBP5’s role in these pathologies provides a new avenue for the development of targeted, non-surgical interventions that could mitigate the adipose overgrowth by modulating the activity or expression of FKBP5. We tested the effects of two FKBP5 inhibitors, SAFit1 and SAFit2, on the adipogenesis of FAPs and found that both drugs effectively inhibited adipogenic differentiation, reducing the expression of adipogenic promoting factors. Currently, SAFit1 and SAFit2 is at a nascent stage, with most studies focusing on their cellular and molecular effects, and have not yet progressed to clinical trials [36]. Translating these findings into practical treatments requires careful balancing act. On one hand, the potential to reduce excessive adipose growth through targeted modulation of FKBP5 is immensely promising; on the other hand, it is crucial to ensure that such interventions do not inadvertently impair the positive roles that adipose tissue and FKBP5 play in the body [24]. Detailed studies to elucidate the precise mechanisms by which FKBP5 influences adipogenesis, and how these can be safely and effectively targeted, will be essential.

This study has certain limitations. It is based on a limited set of samples from FIL patients and control subjects, which restricts the broad applicability and generalizability of the results. A more extensive and diverse sample set might reveal more complex heterogeneity of FAPs and disease-specific molecular markers. Another limitation is the comprehensive interpretation of the pathologic mechanisms. While the study identified an association between PIK3CA variants, FKBP5 expression, and FIL, these mechanisms may only partially explain the pathophysiology of FIL. The pathophysiology of FIL is likely influenced by the interactions of multiple cell types and signaling pathways, including but not limited to immune regulation, collagen deposition, and other factors promoting adipocyte proliferation and differentiation. Our scRNA-seq also identified functional and proportional changes in other cell types (such as immune cells, endothelial cells), but these were not explored in depth in this article, warranting further investigation in subsequent studies.

## Conclusions

Through single-cell sequencing, we discovered that FKBP5 was relatively highly expressed in FIL-FAPs. Subsequent experimental validation demonstrated that FKBP5 was regulated by PI3K-AKT pathways and played a crucial role in regulating the adipogenesis of FIL-FAPs. This study is the first to reveal that FKBP5 can serve as a therapeutic target for FIL.

### Electronic supplementary material

Below is the link to the electronic supplementary material.

**Supplementary Material 1: Table S1.** Clinical information of patients with FIL and patients in control group

**Supplementary Material 2: Table S2.** Primers used for quantitative PCR and lentivirus

**Supplementary Material 3: Table S3.** Prime sequence for shRNA

**Supplementary Material 4: Figure S1.** Expression of selected genes in different clusters. A: Violin plots show the expression levels of different genes across various clusters of FAPs. B: Violin plots display the expression levels of PIK3CA in different clusters within FIL-FAPs and CON-FAPs

**Supplementary Material 5: Figure S2:** Immunofluorescence staining showing the presence of the ASC and PreAs subpopulation in the subcutaneous adipose tissues of FIL patients and healthy donors. A: Staining results in adipose tissue sections of patient with or without FIL. Arrows indicate the PDGFRA^+^ DPP4 ^high^ ASCs cells. B: Arrows indicate the PDGFRA^+^ PRG4 ^high^ ASCs cells. C: Arrows indicate the PDGFRA^+^ APOE ^high^ PreAs cells. Scale bar: 20 μm

**Supplementary Material 6: Table S4:** Differential expression genes in FIL and CON

**Supplementary Material 7: Figure S3:** Characterization of primary FAPs. The expression of the FAPs surface markers CD34, CD90, and PDGFRA, the haematopoietic marker CD45 and the immune marker HLA-DR in isolated FAPs at passage 1 was detected by flow cytometry

**Supplementary Material 8: Figure S4:** Variant information chart for FIL-FAPs from four FIL patients

**Supplementary Material 9: Figure S5:** PIK3CA is essential for adipogenesis of FIL-FAPs in vitro and in vivo. A: Oil Red O staining showed reduced lipid synthesis in PIK3CA-knockdown FIL-FAPs. B: Quantitative assessment of Oil Red O staining area. C: Gene expression analysis examining PPAR γ, C/EBP α, and FABP 4 levels using western blotting in FIL-FAPs and PIK3CA-knockdown FIL-FAPs after adipogenic induction for three days. D: H&E staining of Matrigel implants collected on day 28 and quantification of adipocytes size. E-F: Oil Red O staining of Matrigel implants quantification of Oil red O staining area. G: Oil Red O staining showed increased lipid synthesis in PIK3CA-overexpression CON-FAPs. H: Oil Red O staining showed reduced lipid synthesis in PIK3CA-overexpression CON-FAPs. I: Gene expression analysis examining PPAR γ, C/EBP α, and FABP 4 levels using western blotting in CON-FAPs and PIK3CA-overexpression CON-FAPs after adipogenic induction for three days. J: H&E staining of Matrigel implants collected on day 28 and quantification of adipocytes size. K-L: Oil Red O staining of Matrigel implants quantification of Oil red O staining area. Data were analyzed by one-way ANOVA (B) or Student’s t test (D, F, H, J, L), and are presented as mean ± SD with three replicate experiments (C, I) or three biological replicates (B, D, F, G, J, K). Scale bar: 50 μm. Full-length blots are presented in Additional file 10. Figure S6

Supplementary Material 10

## Data Availability

The datasets used and/or analyzed during the current study are available in NCBI’s Gene Expression Omnibus (GEO) and are accessible through GEO Series accession number: GSE267777.

## Abbreviations

FIL: Facial infiltrating lipomatosis
SVF: Stromal vascular fraction
PIK3CA: Phosphatidylinositol 3-kinase catalytic subunit alpha
FAPs: Fibro-adipogenic precursor cells
FKBP5: FK506 binding protein 51
