# Supplementary material for: Single-cell sequencing of facial adipose tissue unveils FKBP5 as a therapeutic target for facial infiltrating lipomatosis
Source: Stem Cell Res Ther. 2024 Jul 18;15:209. doi: 10.1186/s13287-024-03835-9 (PMC11256636; doi:10.1186/s13287-024-03835-9)
Supplement: Supplementary file 3 — Supplementary Material 3: Table S3. Prime sequence for shRNA [file 13287_2024_3835_MOESM3_ESM.docx]

| **Table S3. Prime sequence for shRNA** | |
| --- | --- |
| Genes | Primer information |

sh-Control 5’-CCTAAGGTTAAGTCGCCCTCG-3’

sh-PIK3CA-1 5’-GCTTGAAGAGTGTCGAATTAT-3’

sh-PIK3CA-2 5’-AGAATATCAGGGCAAGTATAT-3’

sh-FKBP5-1 5’-AAAGTTTATGTCCATTACAAAGG-3’

sh-FKBP5-2 5’-TAAACTTTGTCTCCAATCATCGG-3’

Lv-FKBP5 NM_001145775.3

Lv-PIK3CA NM_006218.4
