# Supplementary figures and images for: Single-cell sequencing of facial adipose tissue unveils FKBP5 as a therapeutic target for facial infiltrating lipomatosis
Source: Stem Cell Res Ther. 2024 Jul 18;15:209. doi: 10.1186/s13287-024-03835-9 (PMC11256636; doi:10.1186/s13287-024-03835-9)

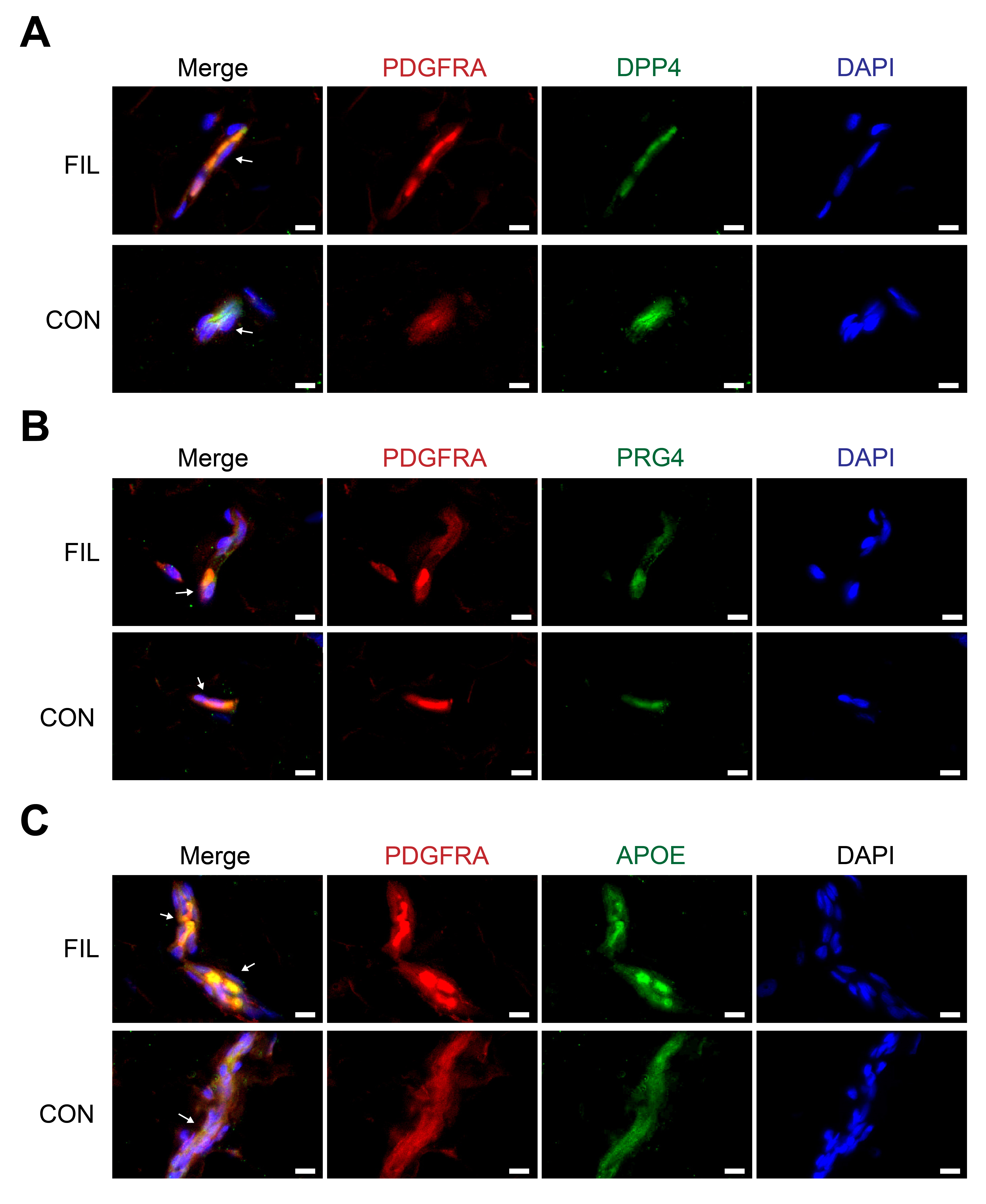

Supplement: Supplementary file 5 — Supplementary Material 5: Figure S2: Immunofluorescence staining showing the presence of the ASC and PreAs subpopulation in the subcutaneous adipose tissues of FIL patients and healthy donors. A: Staining results in adipose tissue sections of patient with or without FIL. Arrows indicate the PDGFRA+ DPP4 high ASCs cells. B: Arrows indicate the PDGFRA+ PRG4 high ASCs cells. C: Arrows indicate the PDGFRA+ APOE high PreAs cells. Scale bar: 20 μm [file 13287_2024_3835_MOESM5_ESM.png]

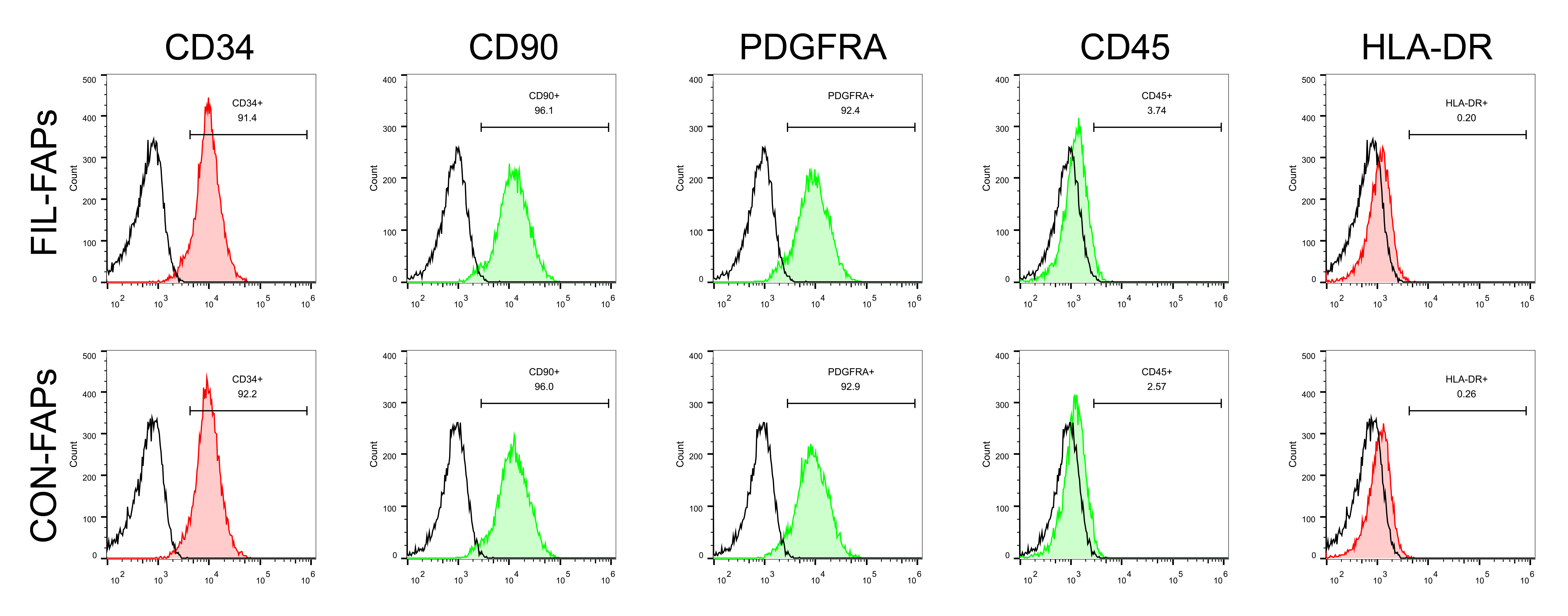

Supplement: Supplementary file 7 — Supplementary Material 7: Figure S3: Characterization of primary FAPs. The expression of the FAPs surface markers CD34, CD90, and PDGFRA, the haematopoietic marker CD45 and the immune marker HLA-DR in isolated FAPs at passage 1 was detected by flow cytometry [file 13287_2024_3835_MOESM7_ESM.jpg]

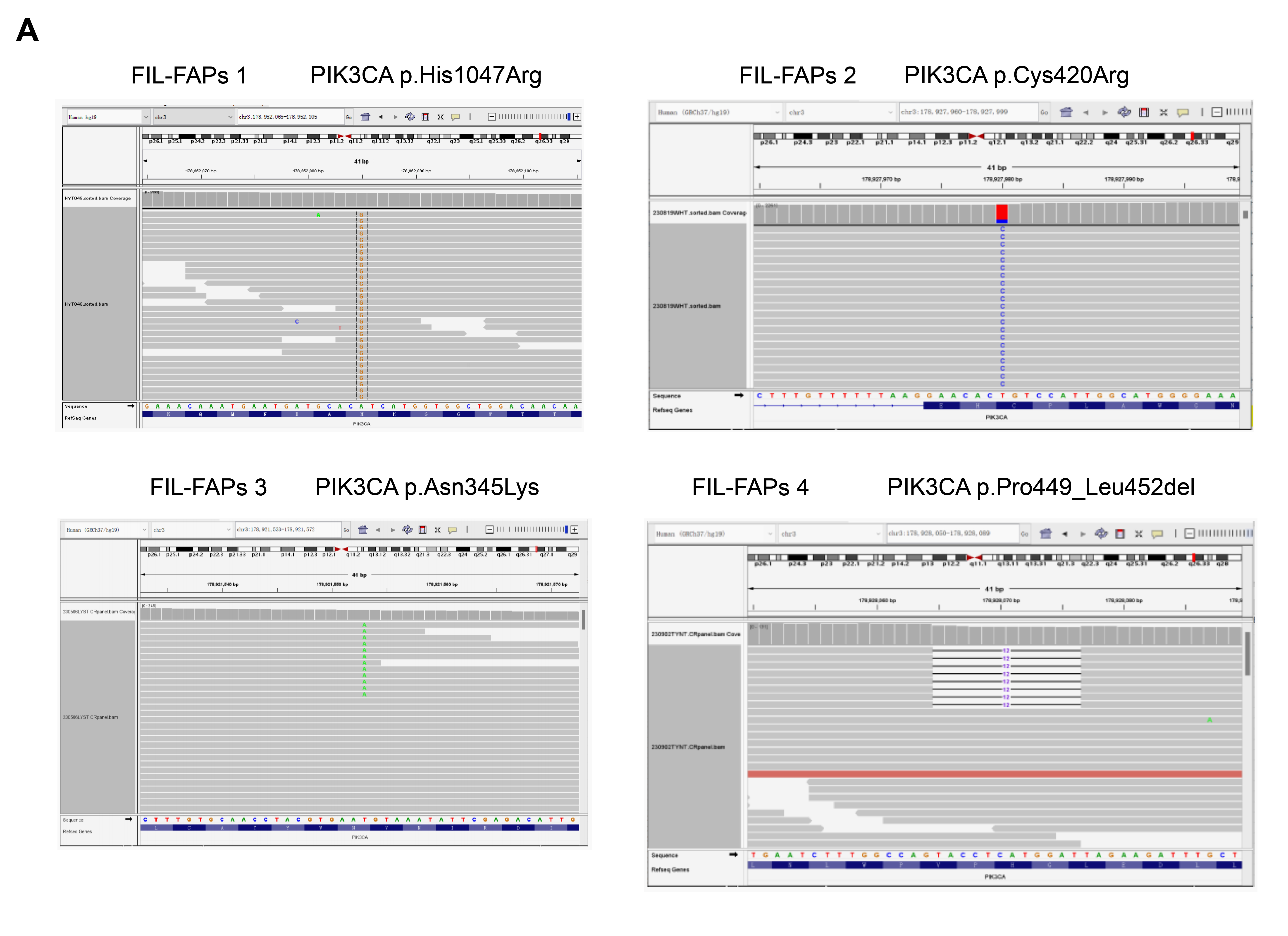

Supplement: Supplementary file 8 — Supplementary Material 8: Figure S4: Variant information chart for FIL-FAPs from four FIL patients [file 13287_2024_3835_MOESM8_ESM.jpg]

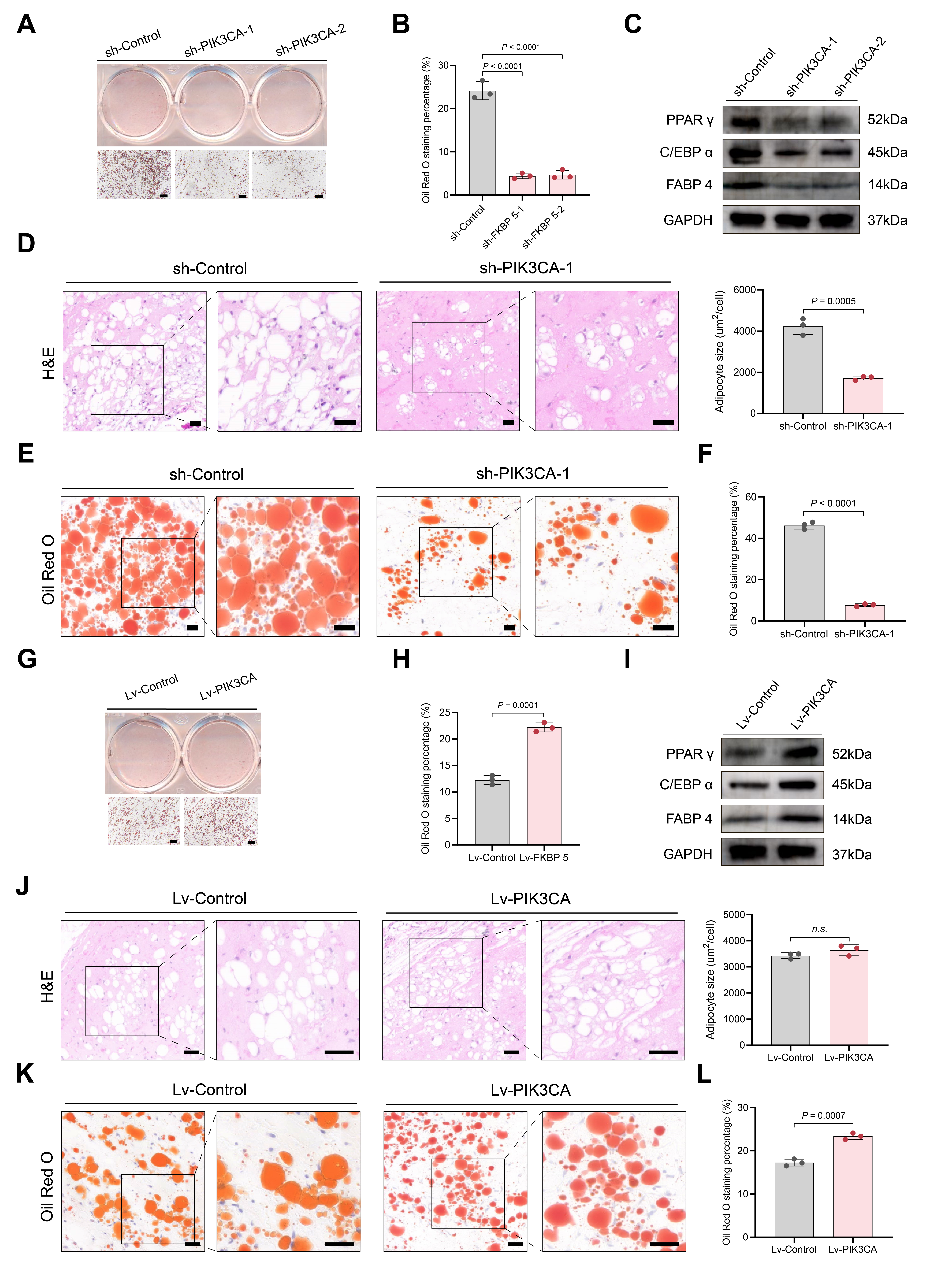

Supplement: Supplementary file 9 — Supplementary Material 9: Figure S5: PIK3CA is essential for adipogenesis of FIL-FAPs in vitro and in vivo. A: Oil Red O staining showed reduced lipid synthesis in PIK3CA-knockdown FIL-FAPs. B: Quantitative assessment of Oil Red O staining area. C: Gene expression analysis examining PPAR γ, C/EBP α, and FABP 4 levels using western blotting in FIL-FAPs and PIK3CA-knockdown FIL-FAPs after adipogenic induction for three days. D: H&E staining of Matrigel implants collected on day 28 and quantification of adipocytes size. E-F: Oil Red O staining of Matrigel implants quantification of Oil red O staining area. G: Oil Red O staining showed increased lipid synthesis in PIK3CA-overexpression CON-FAPs. H: Oil Red O staining showed reduced lipid synthesis in PIK3CA-overexpression CON-FAPs. I: Gene expression analysis examining PPAR γ, C/EBP α, and FABP 4 levels using western blotting in CON-FAPs and PIK3CA-overexpression CON-FAPs after adipogenic induction for three days. J: H&E staining of Matrigel implants collected on day 28 and quantification of adipocytes size. K-L: Oil Red O staining of Matrigel implants quantification of Oil red O staining area. Data were analyzed by one-way ANOVA (B) or Student’s t test (D, F, H, J, L), and are presented as mean ± SD with three replicate experiments (C, I) or three biological replicates (B, D, F, G, J, K). Scale bar: 50 μm. Full-length blots are presented in Additional file 10. Figure S6 [file 13287_2024_3835_MOESM9_ESM.jpg]
