# Supplementary material for: Single-cell sequencing of facial adipose tissue unveils FKBP5 as a therapeutic target for facial infiltrating lipomatosis
Source: Stem Cell Res Ther. 2024 Jul 18;15:209. doi: 10.1186/s13287-024-03835-9 (PMC11256636; doi:10.1186/s13287-024-03835-9)
Supplement: Supplementary file 10 — Supplementary Material 10 [file 13287_2024_3835_MOESM10_ESM.pdf]

Figure 6D

FKBP5

70kDa→
50kDa→

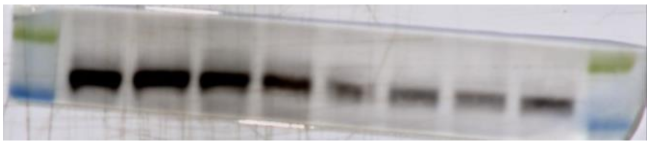

GAPDH

40kDa→
35kDa→

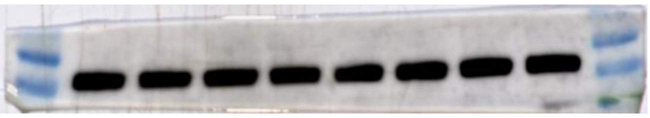

Figure 6E

p110α

130kDa→
100kDa→

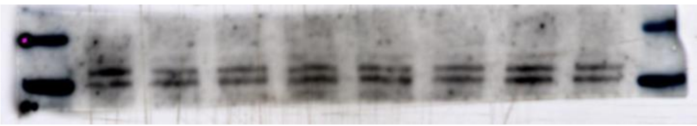

AKT

70kDa→
50kDa→

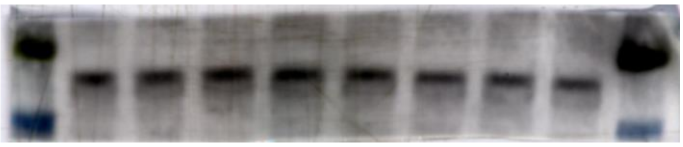

p-AKT

70kDa→
50kDa→

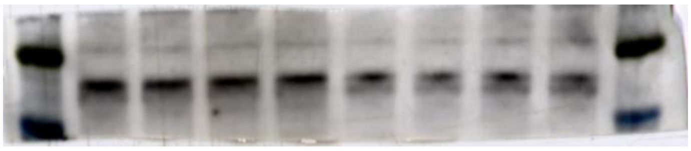

mTOR

300kDa→
250kDa→
180kDa→

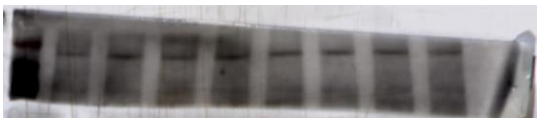

p-mTOR

300kDa→
250kDa→
180kDa→

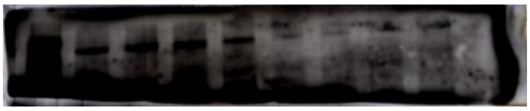

GAPDH

40kDa→
35kDa→

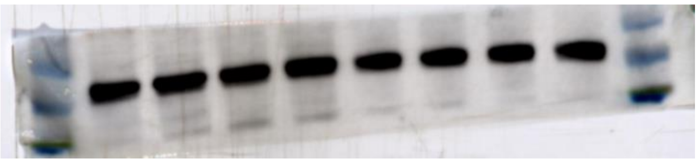

Figure 6G

P110α

130kDa→

100kDa→

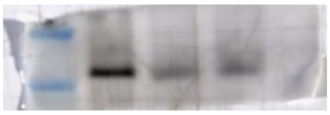

AKT

70kDa→
50kDa→

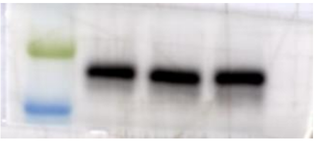

p-AKT

70kDa→
50kDa→

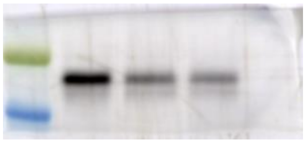

mTOR

250kDa→

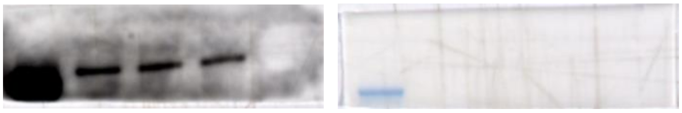

p-mTOR

250kDa→

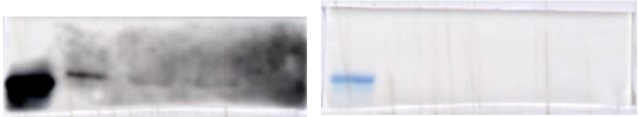

GAPDH

40kDa→
35kDa→

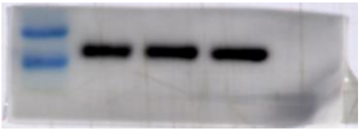

Figure 6I
FKBP5

70kDa→
50kDa→

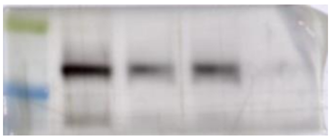

GAPDH

40kDa→
35kDa→

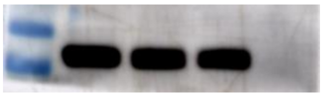

Figure 6K
P110a

130kDa→
100kDa→

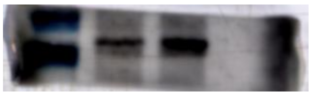

AKT

70kDa→

50kDa→

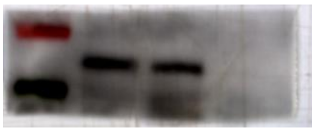

p-AKT

70kDa→
50kDa→

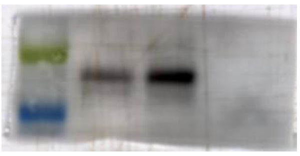

mTOR

300kDa→
250kDa→
180kDa→

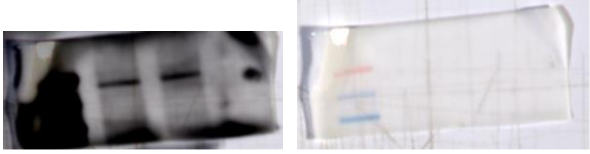

p-mTOR

300kDa→
250kDa→
180kDa→

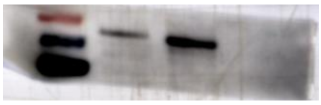

GAPDH

40kDa→

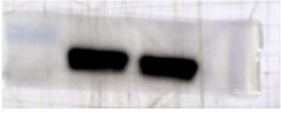

Figure 6M

FKBP5

70kDa→
50kDa→

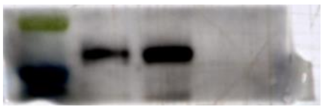

GAPDH

40kDa→
35kDa→

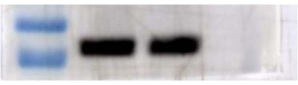

Figure 6N

AKT

70kDa→
50kDa→

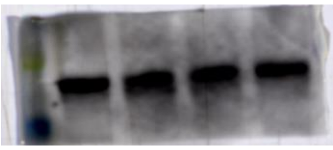

p-AKT

70kDa→
50kDa→

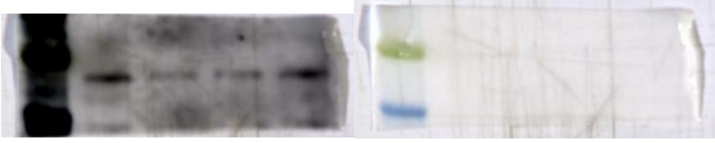

mTOR

250kDa→

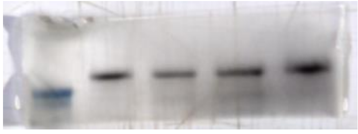

p-mTOR

250kDa→

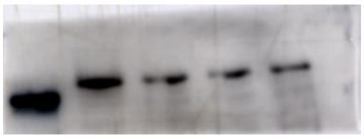

FKBP5

70kDa→
50kDa→

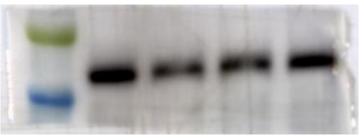

GAPDH

40kDa→
35kDa→

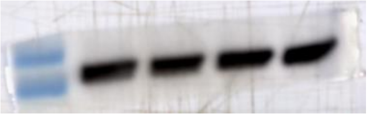

Figure 6O

FKBP5

70kDa→
50kDa→

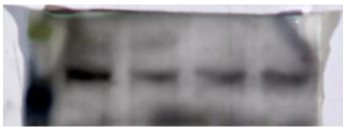

GAPDH

40kDa→
35kDa→

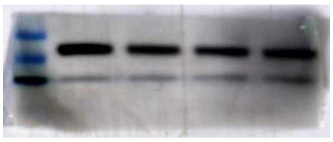

Figure 6P

FKBP5

70kDa→
50kDa→

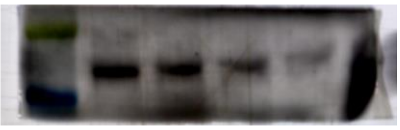

GAPDH

40kDa→
35kDa→

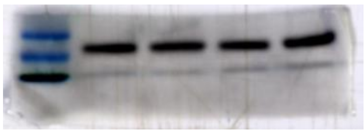

Figure 7B

FKBP5

50kDa→

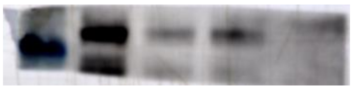

GAPDH

40kDa→
35kDa→

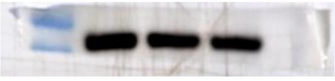

Figure 7E

PPARG

70kDa→
50kDa→

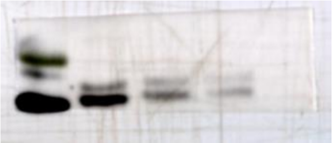

C/EBPA

40kDa→

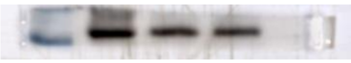

FABP 4

15kDa→

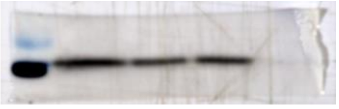

GAPDH

35kDa→

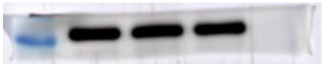

Figure 7G

FKBP5

50kDa→

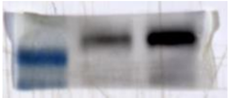

GAPDH

40kDa→
35kDa→

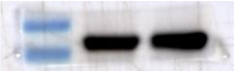

Figure 7I

PPARG

70kDa→
50kDa→

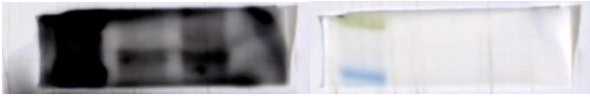

C/EBPA

40kDa→

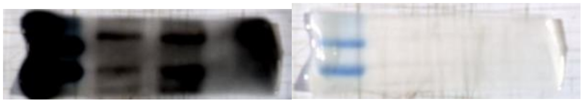

FABP 4

20kDa→

20kDa→

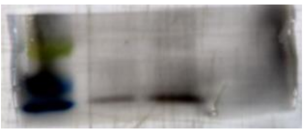

GAPDH

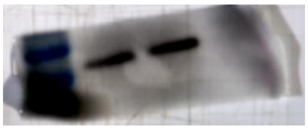

Figure 7L

PPARG

70kDa→
50kDa→

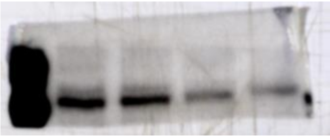

CEBPA

40kDa→

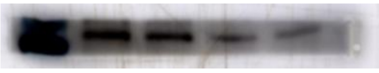

FABP4

20kDa→
15kDa→

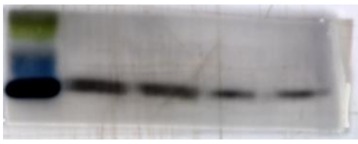

GAPDH

35kDa→

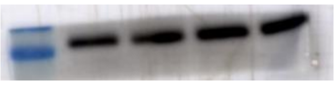

Figure 7N

PPARG

70kDa→
50kDa→

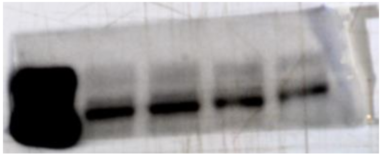

CEBPA

40kDa→

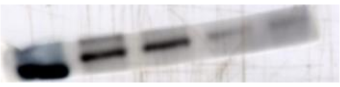

FABP4

20kDa→
15kDa→

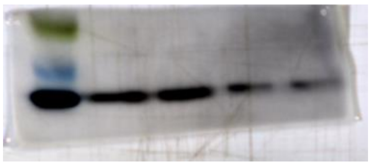

GAPDH

35kDa→

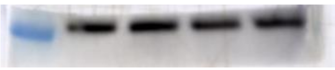

Figure S5C

PPARG

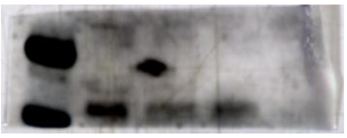

70kDa→
50kDa→

CEBPA

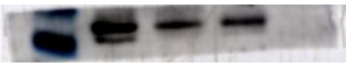

40kDa→

FABP4

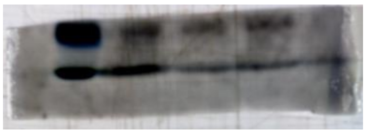

20kDa→
15kDa→

GAPDH

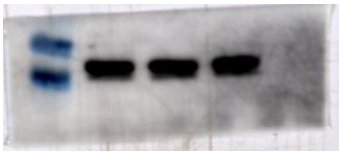

40kDa→
35kDa→

Figure S5I

PPARG

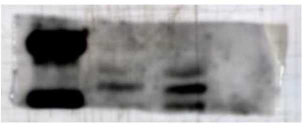

70kDa→
50kDa→

CEBPA

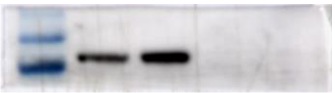

50kDa→
40kDa→

FABP4

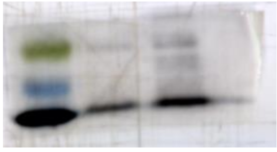

25kDa→
20kDa→
15kDa→

GAPDH

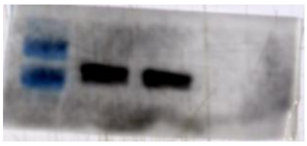

40kDa→
35kDa→
